# Interplay of Energetics and ER Stress Exacerbates Alzheimer's Amyloid-β (Aβ) Toxicity in Yeast

**DOI:** 10.3389/fnmol.2017.00232

**Published:** 2017-07-27

**Authors:** Xin Chen, Markus M. M. Bisschops, Nisha R. Agarwal, Boyang Ji, Kumaravel P. Shanmugavel, Dina Petranovic

**Affiliations:** ^1^Division of Systems and Synthetic Biology, Department of Biology and Biological Engineering, Chalmers University of Technology Gothenburg, Sweden; ^2^Division of Chemical Biology, Department of Biology and Biological Engineering, Chalmers University of Technology Gothenburg, Sweden; ^3^Novo Nordisk Foundation Center for Biosustainability, Chalmers University of Technology Gothenburg, Sweden

**Keywords:** amyloid-β, Alzheimer's disease, energetics, ER stress, yeast

## Abstract

Alzheimer's disease (AD) is a progressive neurodegeneration. Oligomers of amyloid-β peptides (Aβ) are thought to play a pivotal role in AD pathogenesis, yet the mechanisms involved remain unclear. Two major isoforms of Aβ associated with AD are Aβ40 and Aβ42, the latter being more toxic and prone to form oligomers. Here, we took a systems biology approach to study two humanized yeast AD models which expressed either Aβ40 or Aβ42 in bioreactor cultures. Strict control of oxygen availability and culture pH, strongly affected chronological lifespan and reduced variations during cell growth. Reduced growth rates and biomass yields were observed upon Aβ42 expression, indicating a redirection of energy from growth to maintenance. Quantitative physiology analyses furthermore revealed reduced mitochondrial functionality and ATP generation in Aβ42 expressing cells, which matched with observed aberrant mitochondrial structures. Genome-wide expression level analysis showed that Aβ42 expression triggered strong ER stress and unfolded protein responses. Equivalent expression of Aβ40, however, induced only mild ER stress, which resulted in hardly affected physiology. Using AD yeast models in well-controlled cultures strengthened our understanding on how cells translate different Aβ toxicity signals into particular cell fate programs, and further enhance their potential as a discovery platform to identify possible therapies.

## Introduction

Alzheimer's disease (AD) is the most common form of neurodegenerative disorder, and its incidence is projected to rise due to the increasing life expectancy (Wyss-Coray, 2016). Due to incomplete knowledge on the underlying mechanisms that lead to cellular dysfunction in AD, it is difficult to design effective therapies. Accumulation of amyloid-β (Aβ) plaques in the brain is a key neuropathological feature of AD (Hardy and Selkoe, 2002). Aβ peptides (ranging in length from 39 to 43 amino acids) are generated by amyloidogenic processing of the transmembrane amyloid precursor protein (APP; Selkoe, 2001). Differential cleavage of APP produces two major Aβ peptide isoforms, Aβ40 and Aβ42, of which Aβ40 is most abundantly produced, but Aβ42 is the predominant isoform found in AD plaques (Younkin, 1998). Aβ42 is also more hydrophobic and prone to aggregation than Aβ40 (Jarrett et al., 1993). Increasing evidence suggests that oligomeric species of Aβ are the most toxic forms (McLean et al., 1999; Shankar et al., 2008) and the accumulation of intracellular Aβ42 oligomers may be an early event in AD pathogenesis (Gouras et al., 2005).

The strong conservation of the cellular protein quality control system between yeast and human, combined with its genetic accessibility and ease of manipulation and cultivation, make the yeast *Saccharomyces cerevisiae* a powerful model organism to study protein-misfolding pathologies caused by Aβ (Khurana and Lindquist, 2010). Over the past two decades, several humanized yeast models have been developed to study Aβ toxicity (Fruhmann et al., 2017). The earlier models have been successfully used to monitor aggregation patterns of Aβ (Bagriantsev and Liebman, 2006; von der Haar et al., 2007), but failed to recapitulate its toxic effects. More recently developed models illustrated the importance of intracellular trafficking for Aβ toxicity (Treusch et al., 2011; D'Angelo et al., 2013). In neurons, APP is processed to generate Aβ peptides through the secretory pathway as well as the endocytic pathway (Thinakaran and Koo, 2008). This progression through different intracellular organelles and vesicles has been recapitulated in yeast by fusion of the Aβ peptide to secretion signal sequences, i.e., the Kar2 or α-prepro targeting signals (Treusch et al., 2011; D'Angelo et al., 2013). Expression of human Aβ peptides with the ER Kar2 signal ensures that Aβ peptides transit through the secretory pathway and are eventually exported from the cytoplasm. However, the yeast cell wall prevents secreted Aβ from diffusing away, and Aβ peptides re-enter into the cell through endocytosis (Treusch et al., 2011). Similar to observations in human neurons and other AD model organisms (Luheshi et al., 2007), Aβ42 peptides form more oligomers than Aβ40 and exhibit an increased cellular toxicity in yeast (Treusch et al., 2011). These models captured important aspects of Aβ toxicity and identified the yeast homologs of phosphatidylinositol binding clathrin assembly protein (*PICALM*) and other endocytic factors to be involved in Aβ toxicity (Treusch et al., 2011; D'Angelo et al., 2013; Verduyckt et al., 2016).

Although these models capture the cellular trafficking processes essential to Aβ toxicity, the heterologous Aβ expression in yeast is different from native expression in human neurons. In humanized yeast models, expression of Aβ peptides is often under control of a strong inducible promotor, whereas the production in neurons is constitutively. This inducible type of expression allows for well-timed induction of acute cytotoxicity, but is accompanied by a drastic change in carbon-source and hence metabolism, and excludes capturing effects of cellular aging or cumulative effects on toxicity. To avoid these drawbacks, we developed a continuous and tightly regulated Aβ expression model, which mimicked the chronic cytotoxicity that occurs during AD progression better. In our model, we successfully expressed human Aβ peptides (Aβ40 and Aβ42) in a constitutive manner, resulting in a shorter chronological life span (CLS) and increased oxidative stress. Furthermore, strong links between mitochondrial dysfunction and reduced proteasome activity were observed upon mild expression of Aβ42 peptide (Chen and Petranovic, 2015). Here, we take advantage of this improved humanized yeast model to further exploit the effects of Aβ on cellular functioning, viability and energetics following a systems biology approach.

The complexity of AD is illustrated by the continued interplay of unbalanced networks and homeostatic networks in neurons (Castrillo and Oliver, 2015). To capture these dynamics at the molecular and cellular level, time-course analyses of yeast Aβ models are needed. A crucial prerequisite to extract relevant information from the large amount of data obtained (e.g., genome-wide expression profile analysis), is minimization of confounding variables. The widely used shake-flask or tube cultures suffer from several important drawbacks, including lack of online monitoring and continuous control of cultivation parameters (Klöckner and Büchs, 2012), that influence cell growth and physiology and thus hinder data interpretation (Burtner et al., 2011). Therefore, we considered bioreactors an optimal system in which parameters can be continuously monitored and controlled. In addition, analysis of in- and off-gas compositions, aids in evaluating the energetic efficiencies of growth. Monitoring these parameters is relevant for *S. cerevisiae* as a model organism to estimate the interplay of metabolism, energetics, and mitochondrial functions. The typical growth curve consists of an initially predominantly fermentative, fast exponential growth phase (EX) until glucose is exhausted. Then it is followed by slower, fully respiratory growth on ethanol, glycerol, and organic acids during the post-diauxic shift phase (PD). Finally, growth is arrested due to depletion of extracellular carbon sources and the culture enters stationary phase (SP). Transition through these phases heavily depends on culture conditions (Herman, 2002) and strongly influences cell survival during SP (Bisschops et al., 2015). Although bioreactor cultivation offers strong advantages over more simple culture techniques, it is rarely applied in studies of humanized yeast models.

**Figure 1:**
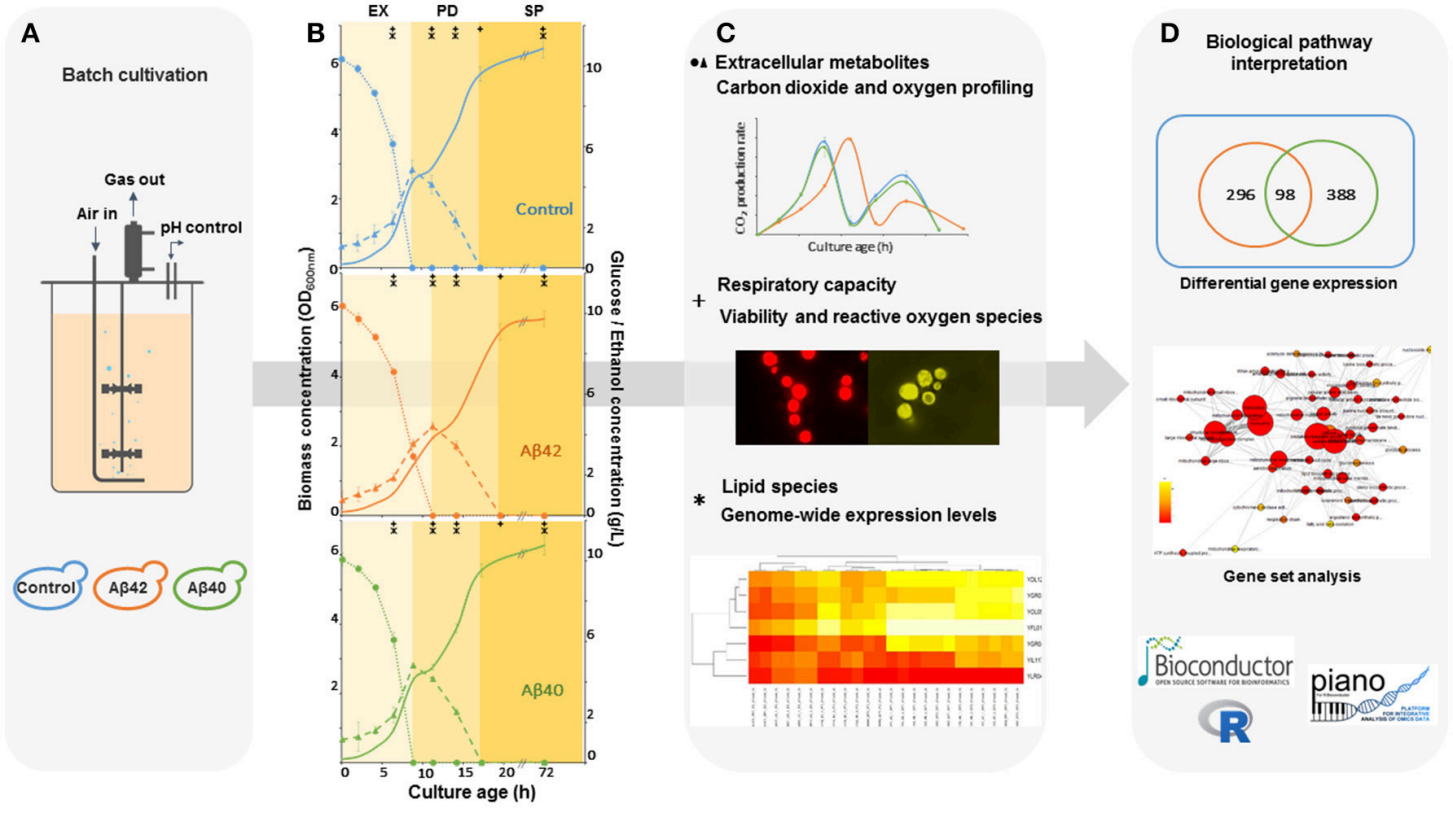
Schematic workflow for the integrated analysis of Aβ-induced cytotoxicity in *S. cerevisiae*. **(A)** Batch cultures of control, Aβ42 and Aβ40 expressing strains were grown and compared in well-controlled bioreactors. **(B)** Biomass, glucose (•), and ethanol (Δ) concentrations were measured and used to divide growth into exponential (EX), post-diauxic shift (PD), and stationary phases (SP). Time-points of specific analyses mentioned in **(C)** are indicated by ^+^ and ^*^. **(C)** Samples were taken for analysis of physiology, lipidome, and transcriptome during different growth phases. **(D)** The data obtained were collected and integrated, resulting in an overall biological interpretation of intracellular Aβ toxicity.

Subjecting our improved humanized Aβ yeast model to strictly controlled and monitored bioreactor environments, allow us, for the first time, to study how cell physiology and genome-wide expression levels are affected by different Aβ variants over time (Figure 1). By controlling the culture parameters, we reduced the number of irrelevant and sidetracking variables and produced a considerable amount of genome-wide and physiological information concerning the energetic consequences of Aβ expression, as well as revealing how these different Aβ toxic isoforms interfered with cellular metabolism and stress response pathways, causing pronounced physiological effects.

## Materials and methods

### Yeast strains and plasmids

The auxotrophic yeast *S. cerevisiae* strain CEN.PK.113-5D (*MATa ura3-52 HIS3, LEU2 TRP1 MAL2-8*^*c*^ *SUC2*; kindly provided by Dr. P. Kötter, University of Frankfurt, Germany; Entian and Kötter, 2007) was used as a host strain in this study. The host strain was transformed with the plasmids for constitutive and equivalent expression of Aβ42 and Aβ40, under control of the *GPD1* promoter: p416GPD-Aβ42 and p416GPD-Aβ40, respectively. The detailed construction of these plasmids and strains has been reported previously (Chen and Petranovic, 2015). Both Aβ constructs consist of the Kar2 signal sequence (42 amino acids) in front of Aβ42 or Aβ40 sequence, as described previously (Treusch et al., 2011).

### Batch cultivation

Strains were grown in defined minimal medium as described previously (Chen and Petranovic, 2015), containing per liter: 10 g glucose, 5 g (NH_4_)_2_SO_4_, 3 g KH_2_PO_4_, 0.5 g MgSO_4_.7H_2_O, 125 μl antifoam 204 (Sigma-Aldrich, USA), trace metals solution, and vitamins. Trace metals and vitamins solutions were prepared as described previously (Jensen et al., 2014). Batch fermentations were performed at 30°C in 1.2-l bioreactors (DasGip, Germany) with a working volume of 700 ml. Cultures were operated with 800 rpm agitation and 1 vvm gas flow of either pure dried air (aerobic) or dried air mixed with nitrogen gas to obtain a mixture containing 2% oxygen (micro-aerobic). Culture pH was measured with a pH sensor (Mettler Toledo, Switzerland) and maintained at 5.0 by automated addition of 2 M KOH or 2 M H_2_SO_4_. The CO_2_ and O_2_ concentrations in the exhaust gas were analyzed real-time with a GA4 gas analyzer (DasGip, Germany).

### Determination of biomass and extracellular metabolites

Biomass and extracellular metabolites were determined as described previously (Zhou et al., 2016). Biomass concentrations were measured as the optical density at 600 nm (OD_600_) and cell dry weight (CDW). CDW was measured by filtering 5 ml of culture samples over a pre-weighted, pre-dried 0.45 μm nitrocellulose filter (Sartorius Stedim, Germany), microwave drying of the filter plus biomass and determining the increase in weight of the dried filter. During the different growth phases, OD/CDW ratios were constant (1.45 ± 0.04 OD/g l^−1^). Extracellular glucose, ethanol, glycerol and acetate concentrations were analyzed on an Ultimate 3000 HPLC (Dionex, Sunnyvale, USA) equipped with an Aminex HPX-87H column (Bio Rad, USA). The column was eluted at 45°C using 5 mM H_2_SO_4_ at a flow rate of 0.6 ml min^−1^.

### Glycogen and trehalose assays

Ten OD_600_ of cells were harvested by centrifugation at 2,000 × *g* for 5 min at 4°C, and washed once in ice-cold distilled water. Cell pellets were resuspended in boiling 0.25 M Na_2_CO_3_ solution and processed as described previously (Parrou and François, 1997). Glycogen and trehalose were converted into glucose with amyloglucosidase (Sigma-Aldrich) and trehalase (Sigma-Aldrich), respectively, in acidic environment (pH 5.2). Glucose levels were determined enzymatically using a Glucose (HK) assay kit (Sigma-Aldrich).

### Viability and reactive oxygen species (ROS) measurement

Viability and intracellular ROS were measured by propidium iodide (PI, Thermo Fisher Scientific, USA) and dihydrorhodamine 123 (DHR123, Sigma-Aldrich, USA) staining respectively, as described previously (Chen and Petranovic, 2015). For the staining, 0.5 OD_600_ of cells were taken at different phases and incubated with 0.5 μg ml^−1^ of PI or 5 μM of DHR123 for 20 min. Cells were analyzed with a Guava flow cytometer (Merck, Germany) using a 488 nm laser for excitation. Fluorescence was detected with a 690/50 filter for PI or a 525/30 nm filter for rhodamine 123. Five thousand cells were analyzed for each sample. Two populations could be distinguished based on fluorescence intensity. Positively stained cells fall within the more fluorescent population (mean fluorescence 5–100 times higher). Results are shown as the fractions stained positively by PI or DHR123.

### Determination of respiratory capacity

Five OD_600_ of cells were harvested, washed twice in distilled water and resuspended in PBS before measurement. Oxygen consumption was measured at 30°C in a 1 ml temperature controlled closed chamber with a Clark oxygen electrode (Gilson) as described previously (Albers et al., 2007). One milliliter of PBS was added to the chamber and incubated until baseline (100% dissolved O_2_) was stable. One OD_600_ of cells were added to the chamber to measure the endogenous oxygen consumption rate. When the rate was stable, 25 mM of glucose was added to the chamber to obtain the initial oxygen consumption rate with glucose. Sodium thiosulfate (NaS_2_O_3_) was used for the zero-point calibration of the sensors. Oxygen consumption rates were determined from the slope of a plot of oxygen concentration vs. time and expressed as mM/OD/h.

### Non-linear microscopy

One OD_600_ of cells were harvested during PD. Mitochondria were stained with 1 μg ml^−1^ Rhodamine 123 (Rh123, Sigma-Aldrich, USA) for 30 min and washed once in PBS. One OD_600_ of cells was harvested during EX for lipid droplets measurement. Non-linear microscopy was applied to detect lipid droplets and mitochondria signals as described previously (Mertz, 2004). Two laser beams, 817 (5 mW) and 1,064 nm (6 mW) of synchronized pico-second pulse trains were spatially and temporally overlapped to be coupled into an inverted Nikon microscope (Eclipse TE2000-E microscope). CARS and TPEF signals were generated in live yeast cells to monitor lipid droplets and Rh123 stained mitochondria, respectively. The laser beams were focused on the samples using a 40x objective (Nikon Plan Fluor N.A. 1.3, working distance 0.21 mm). CARS and TPEF signals were collected in forward and epi-direction, respectively, with a high NA lens with a 661/20 and 514/30 or 609/45 filter into a single photon counting (SPC) photomultiplier tube (HPM-100-40, Becker & Hickl GmbH). Z-stacks were acquired for each sample with 0.2 μm step size for a maximum of 10 μm sample thickness. Each single acquisition in the z-stack was taken in 1 sec with a resolution of 256 pixels for a 34.4 μm field of view. The images were opened in ImageJ and then processed in Imaris (Bitplane software for image processing). The lipid quantification for control and Aβ42 expressing cells was done by ImageJ and GIMP (GNU Image Manipulation Program). Statistics was performed for 10 images of each strain. The quantities were determined from the CARS channel in which lipids were detected. The channel was turned into a binary color format (0 and 1) in GIMP first for lipids and their area in each slice (Li, i = slice number) was calculated using ImageJ. Subsequently cell areas in each slice (Ci, i = slice number) were also calculated. The ratio of the summation of lipid area / summation of cell area gave the value of lipid content in a cell as shown in Equation 1. The distance between slices (Δz) was canceled out since it was constant for all cells.

(1) $$\begin{array}{r} \frac{\sum_{i}Li}{\sum_{i}Ci}=Volume\;of\;lipids\;in\;a\;given\;cell\;volume \end{array}$$

### Transcriptome analysis

Samples for microarray analysis were taken from duplicate cultures during EX, PD, SP1, and SP2, respectively. Samples of cells were frozen rapidly in liquid nitrogen to prevent mRNA turnover (Piper et al., 2002). Total RNA was extracted using the RNeasy Mini Kit (QIAGEN, Germany) with a FastPrep homogenizer (MP Biomedicals, USA) to disrupt cells. Quality of total RNA was assessed by an Agilent 2100 bioanalyzer (Agilent Technologies, USA). Further RNA preparation and hybridization of biotin-conjugated aRNA fragments to Yeast Genome 2.0 Arrays (Affymetrix GeneChip, USA) was performed by the Bioinformatics and Expression Analysis core facility (BEA) of the Karolinska Institute, Sweden. Microarray data are deposited at the Genome Expression Omnibus website (GEO, http://www.ncbi.nlm.nih.gov/geo/) with series number GSE94793. Raw RNA data (CEL files) were preprocessed by Bioconductor and R version 3.2.3. The PIANO package was used for gene ontology (GO) terms and gene set analysis (GSA; Väremo et al., 2013). Only gene sets significantly enriched by distinctly up or down-regulated genes (*p* < 0.05) were considered in this study. The KEGG pathway and Gene Ontology functional categories enrichment analysis were used to investigate the transcriptional regulation of lipogenesis by using online software tool David (Database for Annotation, Visualization, and Integrated Discovery, http://david.abcc.ncifcrf.gov; Huang et al., 2008). Adjusted *P* < 0.05 and fold changes <0.81 or >1.2 were used as thresholds to identify significantly differentially expressed genes. Pheatmap package (cran.r-project.org/web/packages/pheatmap/index.html) was used to generate clustered heatmaps of gene sets.

### Quantitative real-time PCR (qPCR)

qPCR was performed as previously described (Liu et al., 2016). cDNA was synthesized from 1 μg of total RNA using the QuantiTect Reverse Transcription Kit (QIAGEN, Germany). Two microliters of synthesized cDNA were used as the template for qPCR with the DyNAmo Flash SYBR Green qPCR kit (Thermo Fisher Scientific, USA). Housekeeping gene *ACT1* was used as a reference gene to normalize RNA levels. Used primer sets are listed in Table S4.

### Lipid extraction and HPLC-CAD analysis

Lipid extraction and separation were performed as described previously (Khoomrung et al., 2013). Ten micrograms of freeze-dried cells were mixed with 7 ml of chloroform:methanol (2:1, v/v) solution in extraction tubes containing 50 μg of cholesterol as internal standard. Each tube was vigorously vortexed and placed in microwave reaction vessel (12 × 3cm I.D., 0.5 cm thickness, Milestone Stard D, Italy) which was heated to 60°C within 6 min and kept at 60°C for 10 min. After the sample was cooled down to room temperature, 1.7 ml of NaCI was added and the tube was vortexed vigorously. Thereafter, the sample was centrifuged at 1,912 *g* for 10 min and the organic phase was transferred into a clean extraction tube. Organic solvent was evaporated and residues dissolved in 200 μl of chloroform:methanol (2:1, v/v) for HPLC-CAD analysis (Corona, USA). Lipid peaks were identified based on the spectrum of a standard mix (SE, TAG, CH, ES, PA, CL, PE, PC, PS, and PI). Quantification of lipids was performed using serial dilutions of the standard mix from concentrations of 10–1,000 μg ml^−1^. The average log_10_ of peak area was plotted against log_10_ of concentration. Correlation (r^2^) was determined for all standard curves by linear regression.

### Statistical analyses

Significance of differences observed in physiological parameters between strains were determined using two-tailed, student *t*-tests. Cultures were independent replicates and, as parameters were determined using identical procedures, student *t*-tests were performed as two-sample with equal variance. Unless specified explicitly, three independent replicate cultures of the Aβ42 and control strains and two independent replicate cultures of the Aβ40 strain were analyzed under both fully aerated and micro-aerobic conditions. *P* < 0.05 was considered to indicate significant differences. Values are represented as mean ± SEM.

## Results

### Physiological characterization of humanized yeast Aβ strains in controlled bioreactor cultures

To determine the effects of human Aβ peptides on *S. cerevisiae* growth characteristics, strains constitutively expressing either human Aβ42 or Aβ40 peptide (hereafter referred to as Aβ42 and Aβ40 strain) or carrying the empty vector (control strain) were grown in well-aerated bioreactors using glucose as carbon-source (Figures 1A,B). Physiological parameters from these cultures are presented in Table 1. The maximal specific growth rate of the Aβ42 strain was 17% lower than that of the control and Aβ40 strains (*p* < 0.0003), in accordance with our previous report (Chen and Petranovic, 2015). The Aβ42 strain also showed a less pronounced but significantly decreased maximal glucose uptake rate (*p* = 0.02), which was reflected by 15% reduced biomass yield during EX (*p* < 0.02). Maximal ethanol production rates were not significantly altered (*p* > 0.4) during EX. On the contrary, we observed substantially increased production of two fermentation products: glycerol and acetate (Table 1). Glycerol yields were increased in both Aβ strains (*p* < 0.01), but was highest in the Aβ42 strain (2.7-fold compared to control strain, *p* < 0.00005). Despite the well-aerated conditions, the Aβ42 strain also produced more acetate than control and Aβ40 strains (1.28- and 1.21-fold compared to control and Aβ40 strains, respectively, *p* < 0.01). Overall these results suggest an altered redox-cofactor balancing in the heterologous Aβ42 expressing strain (Bakker et al., 2001). During SP, cells rely heavily on the storage carbohydrates glycogen and trehalose as energy sources. No significant differences in the accumulation of glycogen were observed among these strains (Figure S1A). Levels of trehalose were significantly higher in the Aβ42 strain during PD and SP1 (when extracellular carbon source was depleted; Figure S1B), which may be linked to its alternative role in stress-resistance. Trehalose can stabilize proteins in their native folding during heat shock and reduce the aggregation of denatured proteins in yeast cells (Singer and Lindquist, 1998). Reduced viable cell fractions might explain reduced growth and substrate consumption rates in the Aβ42 strain. However, viability analysis showed that fractions of dead cells were low for all strains during different phases (<2.5%). The slightly, yet significantly, elevated fractions of dead cells in Aβ42 cultures (*p* < 0.007, Figure S2A), did not fully explain the observed differences in fermentation kinetics.

**Table 1:** The physiological parameters of all strains during aerobic batch cultivation.

**Strain**	**μ_max_^a^(/h)**	**r_glucose,max_^b^(g/g/h)**	**Y^c^_X/S_ (g/g)**	**Y^d^_glycerol/S_ (mCmol/Cmol)**	**C^e^_acetate,max_ (mmol/L)**	**r^f^_ethanol,max_ (g/g/h)**	**Respiratory quotient^g^**	**r^h^_O2,max_ (mmol/g/h)**
Control	0.368 ± 0.002	2.34 ± 0.05	0.39 ± 0.01	13.5 ± 1.5	3.6 ± 0.2	0.24 ± 0.02	0.42 ± 0.01	8.2 ± 0.5
Aβ40^i^	0.366 ± 0.004	2.27 ± 0.09	0.40 ± 0.03	20.4^*^ ± 0.5	3.8 ± 0.2	0.25 ± 0.01	0.44 ± 0.01	7.4 ± 0.2
Aβ42	0.304^*^ ± 0.003	2.18^*^ ± 0.05	0.36^*^ ± 0.01	35.3^*^ ± 1.4	4.6^*^ ± 0.1	0.22 ± 0.01	0.40 ± 0.02	7.5 ± 0.2

Values are represented as the average of three independent biological replicates ± SEM. The asterisk (

* *) indicates significant different values from the control strain parameters (p < 0.05)*.

a Maximal biomass-specific growth rate on glucose;

b Maximal biomass-specific glucose uptake rate;

c Final biomass yields on substrate;

d Glycerol yields on glucose;

e Maximal acetate concentrations;

f Maximal biomass-specific ethanol consumption rate during PD;

g Respiratory quotient, the ratio of carbon dioxide produced over oxygen consumed during PD;

h Maximal biomass-specific oxygen uptake rate during PD;

i *Average values from biological duplicates*.

### Aβ42 expression strongly affects mitochondrial functionality

**Figure 2:**
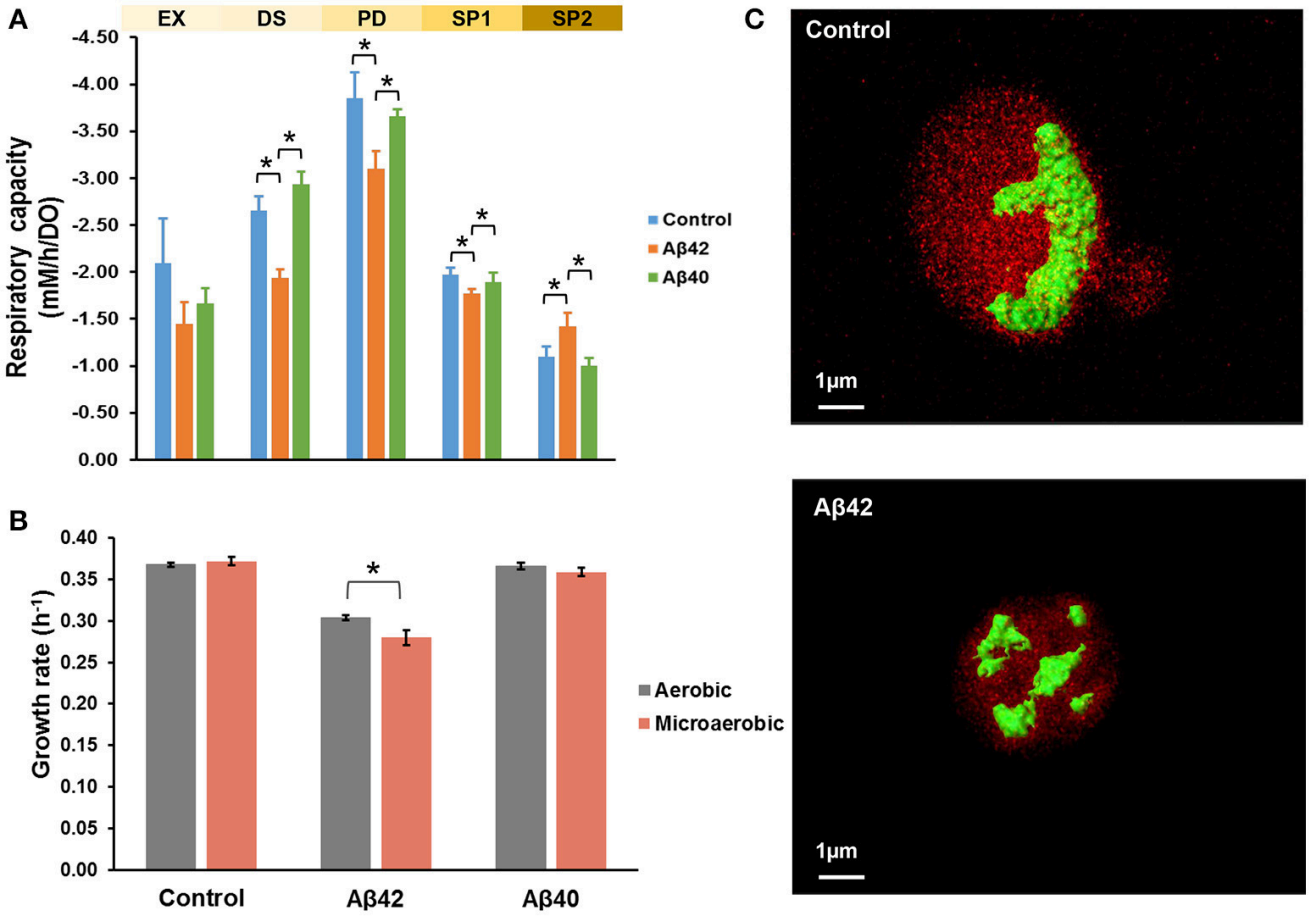
Aβ42 expression causes abnormal mitochondrial functionality and morphology. **(A)** Respiratory capacity was measured during EX, diauxic shift (DS), PD, early SP (SP1), and late SP (SP2) phases. Measurements were performed in duplicate (Aβ40) or triplicate (control and Aβ42) during EX, DS, SP1, and SP2 phases. During PD phase, measurements were done in quadruplicate (Aβ40 strain) or sextuplicate (control and Aβ42 strains), when cells solely relied on respiration. **(B)** Comparison of growth rates between aerobic and microaerobic conditions in bioreactors. **(C)** TPEF microscopy images of representative mitochondrial morphology in PD from control and Aβ42-expressing cells. Mitochondria were stained by Rhodamine 123 and are shown in green. The background fluorescence of cells is shown in red and is indicative of the cell volume. Data shown are average values ± SEM, of triplicate (Aβ42 and control) or duplicate (Aβ40) independent biological replicates. The asterisk (^*^) indicates significant differences (*p* < 0.05).

Mitochondria are pivotal to the survival of cells, including neurons, due to their role in energy metabolism. The increased glycerol production in the Aβ42 strain suggested that the surplus of NADH produced in anabolism, could not be completely reoxidized by mitochondrial respiration (Table 1). Despite the differences in glycerol and acetate production, the maximal oxygen uptake rates during the fully respiratory PD phase were surprisingly similar for both Aβ strains, yet lower than control strain (Table 1). These observations suggested that not consumption of oxygen *per se*, but rather its usage was affected specifically by Aβ42 expression. Together with our previous observations that the respiratory rate of the Aβ42 strain was significantly reduced in ethanol-grown cultures (Chen and Petranovic, 2015), this suggests that the toxicity triggered by Aβ42 might involve reduced mitochondrial functionality. To further test this, we evaluated the respiratory capacity of all strains during different growth phases. During EX no significant differences were observed between strains, which matched with fermentation being the main catabolic pathway at this phase. However, during the subsequent phases (PD and SP1), the respiratory capacity of the Aβ42 strain was significantly decreased compared to both other strains (*p* < 0.05, Figure 2A). Impaired mitochondrial function can lead to excess reactive oxygen species (ROS) production which contributes to AD pathology (Livnat-Levanon et al., 2014). In the Aβ42 strain, the ROS-positive fractions were significantly increased during phases of high metabolic activity, i.e., EX and PD, compared to the other strains (*p* < 0.003, Figure S2B). To investigate if these physiological indications of mitochondrial dysfunction coincided with aberrant mitochondrial structures, we stained the mitochondria with Rhodamine 123 and employed TPEF (two-photon excited fluorescence) microscopy to investigate the morphology in fully respiratory growing cells. Control cells exhibited continuous mitochondrial structures (networks), whereas Aβ42 expressing cells displayed a more fragmented structure of mitochondria (Figure 2C and Videos S1, S2). Such fragmented structures of mitochondria were previously observed in aged yeast cells and could enhance the turn-over of damaged mitochondria by mitophagy (Mao and Klionsky, 2013; Breitenbach et al., 2014).

### Oxygen-limited conditions enhance impact of Aβ42 expression on physiology

The affected mitochondrial functionality is likely to have a more severe impact on cells under oxygen-limited conditions. Oxygen limitation is common due to increased oxygen-requirements during PD in less-controlled cultivations, such as shake-flasks, in which oxygen-transfer rates are lower (Anderlei and Büchs, 2001). We also noticed this effect by the differences in viability of the same Aβ42 strain reported here (Figure S1) and previously in shake-flasks (Chen and Petranovic, 2015). In standard bioreactor cultures, the dissolved oxygen levels were maintained above 30% of saturation at all time, but to test the combinatorial effect of oxygen limitation and Aβ peptides expression on cell physiology, oxygen-limited cultivations were also performed. To this end, the oxygen concentration in the inflowing gas was reduced to 2%, leading to dissolved oxygen levels below 1% of saturation. The reduced dissolved oxygen levels only reduced the maximal growth rate of the Aβ42 strain on glucose significantly (8% reduction, *p* < 0.05) and did not significantly affect growth of control and Aβ40 strains (Figure 2B). Similarly, total biomass yields were only significantly reduced in the Aβ42 strain (13% reduction, *p* = 0.05; Table S1). Under well-aerated, standard conditions, we observed differences in maximal oxygen uptake rates between Aβ42 and control strains (Table 1). When the oxygen supply was severely limited, oxygen uptake rates were also restricted and differences between strains were absent (Table S1). However, despite these equal oxygen consumption rates, the Aβ42 strain produced less carbon dioxide as shown by the almost one-third reduction in respiratory quotient (defined as the ratio of carbon dioxide produced/oxygen consumed) compared to control strain (*p* < 0.05, Table S1). This indicates an altered usage of the oxygen consumed by the Aβ42 strain, likely due to less efficient respiration. In addition to these effects on physiology, oxygen limitation also resulted in a doubling of the fraction of dead cells in all strains (<6%; Figure S2A). The ROS-positive fractions were similar compared to well-aerated conditions for all strains during EX and PD, but lower during SP, likely due to reduced oxygen availability (Figure S2B).

Oxygen limitation may thus contribute to enhanced cell death, however the drastic effect of Aβ42 expression on CLS observed in shake-flask cultures (Chen and Petranovic, 2015) was not observed in oxygen-limited bioreactor cultures. Only 5.25 ± 0.44 and 2.39 ± 0.64% of cells were identified as dead in Aβ42 and control cultures, respectively, during SP2, i.e., 2 days after extracellular carbon source exhaustion (Figure S2A), in contrast to 37.15 ± 1.21 and 14.06 ± 1.82%, respectively, in shake-flask cultures (Chen and Petranovic, 2015). A putative explanation could be the different glucose concentrations in the medium used for bioreactors and shake-flask cultivations, i.e., 10 g L^−1^ instead of 20 g L^−1^, respectively. Shake flask cultures grown in medium containing 10 g L^−1^ of glucose which is identical to the medium used in bioreactor cultures, revealed indeed a strong effect on viability. The higher initial glucose concentration resulted in a strongly reduced CLS, i.e., the fractions of dead cells were 5–7 fold higher during SP2 for the different strains (Figures S3A,B). Both initial glucose concentrations are generally not considered to induce calorie restriction related hormesis effects, but strongly affect other culture parameters in stationary phase. When using 10 g L^−1^ of glucose the pH dropped to only 4.5, instead of to 3.1, when 20 g L^−1^ of glucose was used (Figures S3C,D). Final cell concentrations correlated with initial glucose concentrations (Figures S3E,F). The acidic environment poses a strong stress on cells and can result in reduced CLS. An additional confounding factor were higher acetate levels in shake-flask cultures, which were highest for the Aβ42 strain on 20 g L^−1^ glucose (Figure S4). Altogether oxygen limitation, low pH-values and high acetate concentrations may contribute to the strongly reduced viability and CLS of all strains and especially of the Aβ42 strain in less-controlled shake-flasks environment. These effects are smaller in bioreactor cultures, because oxygen is not limited and a decrease in pH is prevented by online-monitoring and automated titration of base.

### Global transcriptional response to Aβ expression

**Figure 3:**
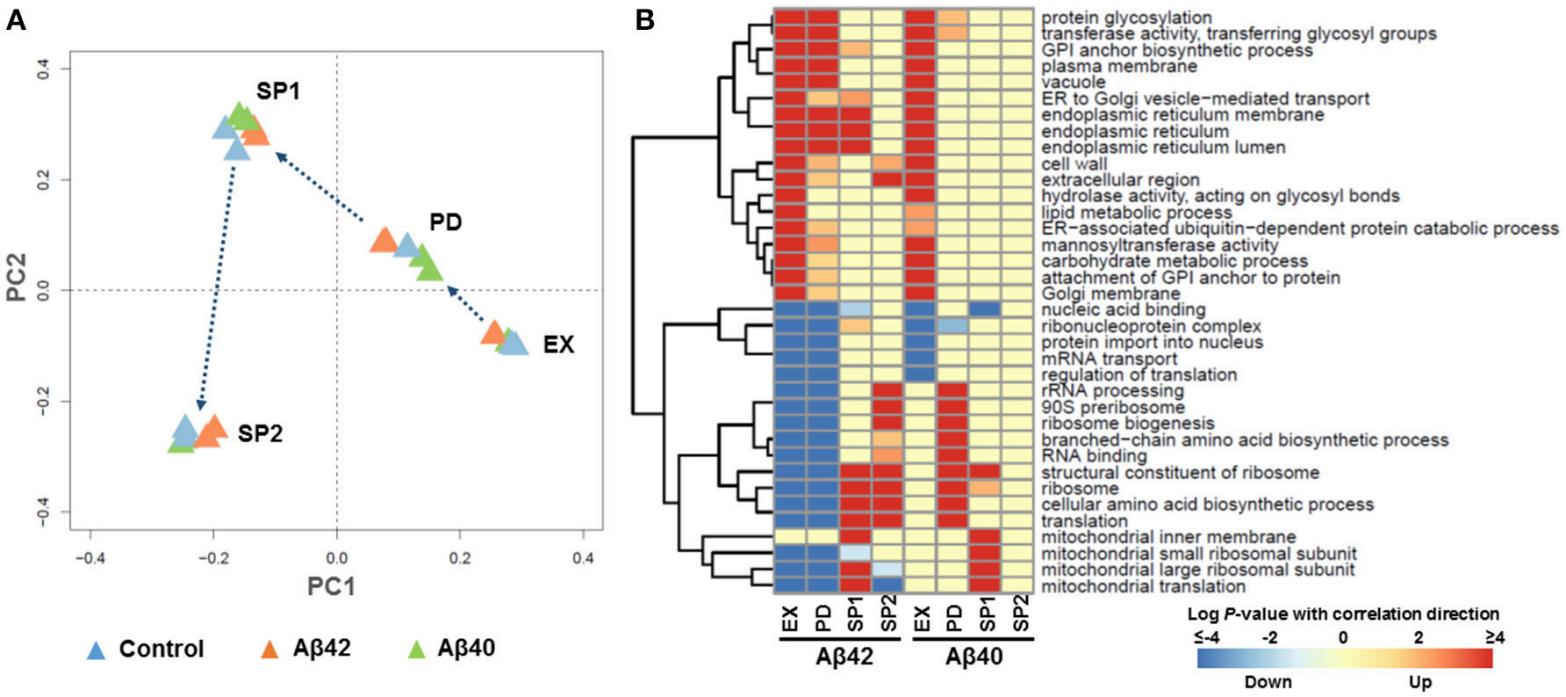
Global transcriptional response to Aβ expression. **(A)** Principle Component Analysis (PCA). Each triangle represents one biological replicate. **(B)** The most strongly enriched GO terms of biological processes in Aβ42 and Aβ40 strains, compared to the control strain (*p* < 0.005) during EX, PD, SP1, and SP2, with red color indicating up-regulated processes and blue indicating down-regulated processes. For the complete gene set analysis results, see Figures S7, S8.

To understand the mechanisms behind the phenotypic changes in Aβ42 and Aβ40 strains, gene expression levels during EX, PD, SP1, and SP2 were quantified using microarrays. The global expression pattern was characterized using principal component analysis (PCA). PCA resulted in strong grouping of biological replicates, indicative of a high degree of reproducibility. The first and second PCA components clearly separated all of samples from different growth phases by their gene expression profiles (Figure 3A and Figure S5). To further examine the extent of changes in each phase, we performed pair-wise comparisons between the strains at each time-point. The expression of 472, 394, 391, and 280 genes was significantly different (adjusted *p* < 0.001) during EX, PD, SP1, and SP2, respectively, between the Aβ42 strain and the control strain (Figure S6A). Although the physiology of the Aβ40 strain was not notably different from the control strain, expression of 89, 486, and 1,118 genes was significantly different (adjusted *p* < 0.001) during EX, PD, and SP1, respectively. No significantly changed genes were identified later in SP2 between Aβ40 and control strains (Figure S6B). Of note, the overlap in differentially expressed genes was <20% between Aβ42 and Aβ40 strains during all phases, suggesting that different biological processes were influenced (Figure S6C).

To gain more insight in biological processes affected by Aβ42 and Aβ40 expression, gene set analysis (GSA) was performed on the significantly differentially expressed genes. Significant enrichment of 126 and 129 gene sets was identified (*p* < 0.005) among genes differentially expressed in Aβ42 and Aβ40 strains, respectively compared to the control strain. The complete list of gene sets can be found in Figures S7, S8. The most significantly enriched gene sets are shown in Figure 3B for both Aβ strains. Gene sets associated with protein processing (post-translational modifications, transport, and degradation), such as, “endoplasmic reticulum (ER),” “protein glycosylation,” “vacuole” were enriched with up-regulated genes during EX, PD, and SP1 in the Aβ42 strain, but only during EX in the Aβ40 strain. Gene sets related to protein synthesis such as, “ribosome,” “translation,” “mitochondrial translation,” and “cellular amino acid biosynthetic process,” were enriched with down-regulated genes in the Aβ42 strain during EX and PD. Remarkably, these genes were up-regulated in the Aβ42 strain compared to the control strain during SP. The expression profiles of these protein synthesis related genes were studied throughout the different culture phases. This revealed that for genes, whose expression was higher in the Aβ42 strain than control strain in SP, this was due to a less pronounced decrease in expression (data not shown). These protein synthesis-related gene sets were enriched with up-regulated genes in the Aβ40 strain especially in PD. This opposite regulation of these genes clearly indicated different responses to Aβ42 and Aβ40 expression. For illustration, the genes involved in amino acid biosynthesis pathways of which expression levels were significantly changed can be found in Figure S9 and Table S2. In addition to these processes directly involved in protein synthesis and processing, transcription related gene sets such as, “nucleic acid binding,” “protein import into nucleus,” “ribonucleoprotein complex,” and “mRNA transport” were enriched with down-regulated genes in both Aβ strains.

### Aβ42 expression induces strong ER stress response

**Figure 4:**
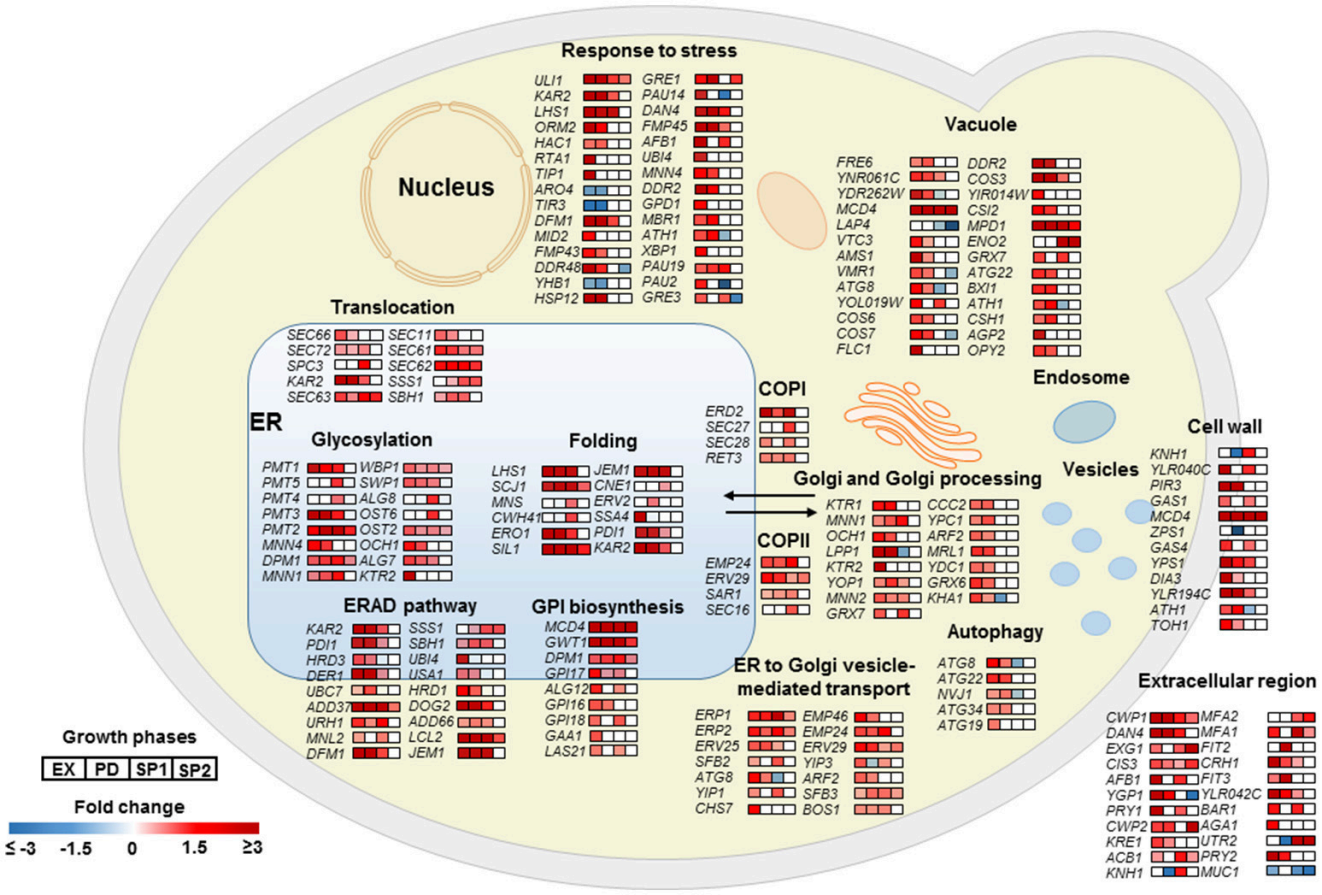
Transcriptional profiles of differentially expressed genes encoding proteins involved in protein secretory and metabolic processes in the Aβ42 strain. Changes in gene expression are shown as fold changes compared to the control strain during EX, PD, SP1, and SP2.

As a key component of cellular proteostasis, the ER is responsible for processing of one-third of cellular proteins and harbors an elaborate protein quality control system (PQC) to eliminate misfolded proteins by degradation (Cao and Kaufman, 2012). However, an overload of the PQC machinery results in an ER stress response (ESR) and activates the unfolded protein response (UPR; Scheper and Hoozemans, 2015). In the Aβ42 strain, most genes involved in “response to stress” were significantly higher expressed, including *HAC1*, the key regulator of UPR. In response to ER stress, *HAC1* (*HAC1*^*u*^) mRNA is spliced to *HAC1* (*HAC1*^*s*^) to initiate synthesis of the active transcription activator Hac1p which induces expression of over 300 of UPR target genes (Travers et al., 2000). The ratio of *HAC1*^*s*^/*HAC1*^*u*^ was 19, 17, and 13-fold higher in the Aβ42 strain compared to the control strain during EX, PD, and SP1, respectively (Figure S10A). Induction of UPR target genes results in the biosynthesis of chaperones, and other factors involved in the secretory pathway, and ER associated degradation (ERAD) to restore ER homeostasis. The gene sets related to protein processing were strongly triggered upon expression of Aβ42 (Figure 4). Genes encoding “folding” chaperones such as, *KAR2, SIL1, SCJ1, JEM1*, and disulfide bond formation enzymes *PDI1* and *ERO1* were indeed all found significantly up-regulated (Figure 4). Higher transcription levels of *PDI1* and *ERO1* were verified by qPCR (Figures S10B,C). Genes encoding proteins involved in processes throughout the secretory pathway including translocation, glycosylation, GPI (Glycosylphosphatidylinositol) biosynthesis, trafficking between ER and Golgi, ER associated degradation (ERAD), and vacuole were also significantly higher expressed in the Aβ42 strain (Figure 4). Besides ERAD, which is the predominant cellular mechanism to degrade misfolded proteins under ER stress, autophagy can also be activated when the amount of misfolded proteins exceeds the ER capacity (Bernales et al., 2006). In line with this, the Aβ42 strain expressed the autophagy related genes *ATG8, ATG22, ATG34, NVJ1*, and *ATG19* to a higher extent (Figure 4).

In the Aβ40 strain, changes in expression levels of genes involved in protein processing were less pronounced (Figure S11). The ratio of *HAC1*^*s*^/*HAC1*^*u*^ in the Aβ40 strain was only 5.8-fold higher than control strain in EX. qPCR confirmed that transcript levels of *PDI1* and *ERO1* were not significantly changed (Figure S10). The distributions of significantly differentially expressed genes between Aβ42 and Aβ40 strains are shown in Figure S12 (*p* < 0.05). All differentially expressed genes involved in protein processing are listed in Table S3.

### Aβ42 expression increases lipid synthesis

**Figure 5:**
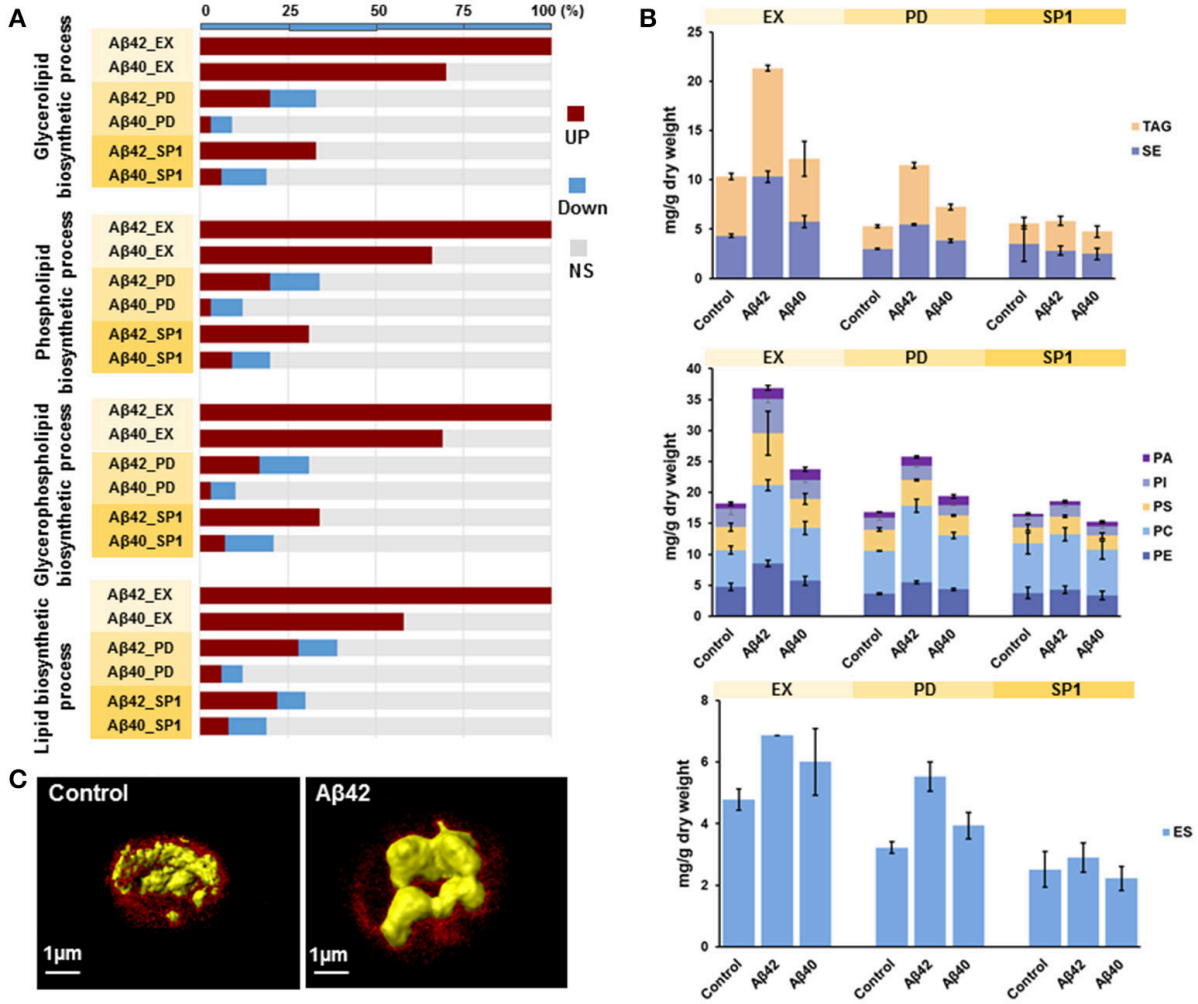
Aβ42 expression results in increased lipid synthesis. **(A)** For significantly enriched GO terms involved in lipid biosynthetic processes, the percentages of genes that are either higher (red), lower (blue), or not significantly (NS, gray) differentially expressed in both Aβ strains during EX, PD, and SP1 are shown. **(B)** Cellular concentrations of storage lipids, phospholipids and ergosterol were measured by HPLC-CAD in all strains. TAG, Triacylglycerols; SE, steryl esters; PA, phosphatidic acid; PI, phosphatidylinositol; PS, phosphatidylserine; PC, phosphatidylcholine; PE, phosphatidylethanolamine; ES, sterol ergosterol. **(C)** CARS microscopy visualization of lipid droplets (in yellow) during EX in control and Aβ42-expressing cells. The background fluorescence of cells is shown in red and is indicative of the cell volume.

It was previously shown that ER stress and activation of UPR pathways play a critical role in lipid metabolism in different model organisms (Hetz, 2012). GSA revealed that the gene sets related to “lipid metabolic process” were overrepresented with up-regulated genes in both Aβ strains during EX. More specifically, gene sets involved in “glycerolipid biosynthesis process,” “phospholipid biosynthetic process,” “glycerophospholipid biosynthetic process,” and “lipid biosynthetic process” were strongly overrepresented among up-regulated genes in the Aβ42 strain during EX, compared to the control strain. Similar results were also found for the Aβ40 strain. However, for the Aβ40 strain, the enrichment was less strong and the amounts of significantly up-regulated genes involved in each processes were, respectively, 30, 34, 31, and 42% lower than for the Aβ42 strain (Figure 5A). The expression level of *INO1*, the gene encoding an important regulator in lipid metabolism in yeast (Henry et al., 2012), was significantly increased in the Aβ42 strain during EX (data not shown). To see whether the different expression patterns led to alterations in cellular lipid composition, we measured different lipid classes dynamically in all three strains. The major lipid constituents of *S. cerevisiae*: storage lipids, phospholipids, and sterols were analyzed. Compared to the control strain, the levels of all lipid categories were significantly increased during EX and PD in the Aβ42 strain. The differences were most pronounced during EX. In the Aβ40 strain, only phospholipids showed a slight but significant increase during EX and PD compared to control strain (Figure 5B). To gain insight in the structural storage of lipids we monitored the three-dimensional distribution and amounts of lipids stored at the single-cell level (which usually coalesce into lipid droplets) by CARS (coherent anti-Stokes Raman scattering) microscopy in exponentially growing cells. CARS can probe and image lipid structures by vibrations between carbon and hydrogen bonds and hence no fluorescent labels or tags are needed. The average lipid droplet volume was 1.6-fold larger in the Aβ42 strain (0.078 ± 0.032 μm^3^) than in the control strain (0.048 ± 0.028 μm^3^; Figure S13). CARS images showed that the lipid droplets in control cells were numerous and smaller in sizes, whereas they tended to accumulate and form larger drops in Aβ42 expressing cells (Figure 5C and Videos S3, S4).

## Discussion

Humanized yeast models have been exploited to investigate protein functions and cellular pathways implicated in neurodegenerative disorders including Huntington's disease (Giorgini et al., 2005), Parkinson's disease (Outeiro and Lindquist, 2003), and AD (Zhang et al., 1994). To study the cytotoxicity of Aβ peptides in AD, several yeast models have been explored (Verduyckt et al., 2016), of which only recent models recapitulate important aspects of Aβ cytotoxicity by including intracellular trafficking (Treusch et al., 2011; D'Angelo et al., 2013). To overcome a major disadvantage of these models, i.e., the use of inducible promoters resulting in strong acute cytotoxicity, we recently presented an improved yeast model in which human Aβ peptides are constitutively expressed, only moderately affecting growth. To our knowledge all studies with these and similar models have been carried out in uncontrolled cultivation systems, such as, shake-flask, tubes, or 96-well plates due to their ease and scalability. However, these cultures present a highly dynamic environment, with changes in nutrient and metabolite levels, pH, and oxygen availability (Büchs, 2001), that strongly influence the growth of Crabtree-positive *S. cerevisiae*. In contrast, the stable (controlled) environments in bioreactor cultures are well defined and highly reproducible. In this paper, we dynamically elucidate the effects of different human Aβ peptides expression on yeast physiology and transcriptome by using bioreactor cultures. This allowed partial confirmation of earlier observation on the role of Aβ peptides in AD pathology (Treusch et al., 2011; Nair et al., 2014; Chen and Petranovic, 2015), but also revealed remarkable differences and increased our knowledge on the mechanistic principles behind cytotoxicity of different Aβ peptides.

Both shake-flask and bioreactor cultures displayed a reduction in the maximal growth rate of the Aβ42 strain (Chen and Petranovic, 2015). However, the previously observed drastic effect on CLS was significantly reduced in bioreactor cultures, which underlines the importance of culture conditions in aging and aging-related disease models (Longo et al., 2012). There may be several reasons for the observed difference in CLS between the two experimental set-ups. First and likely foremost, the well-controlled environment reduced additional stresses on cells. As illustrated by the comparison of aerobic and micro-aerobic conditions in bioreactor cultures, sufficient availability of oxygen had a beneficial effect on CLS (Figure S2A). An additional important parameter kept constant in bioreactor cultures was pH. Neuronal excitability is highly susceptible to fluctuations of intracellular and extracellular pH (Ruffin et al., 2014). One of the main clinical presentations of AD includes signs of decreased brain pH (Demetrius and Simon, 2012). The combination of low pH and presence of toxic peptides might contribute to neuronal cell death. As we confirmed in our yeast model, in non-pH-controlled shake-flask cultures, the acidity of medium increases due to both ammonium consumption and the production of acids, such as acetic acid and CO_2_ (Fraenkel, 1982; Burtner et al., 2009). The degree of acidification depends on medium composition and strain. In our study, an increase in initial glucose concentration from 10 to 20 g L^−1^ resulted in a more than 10-fold increase in proton concentration (Figures S3C,D). Virtually all cellular processes are dependent on intracellular pH (Orij et al., 2012), for example acidification of the cytosol is an early event to regulate caspase-dependent apoptosis in yeast (Matsuyama et al., 2000), and collapse of intracellular pH homeostasis shifts the model of cell death from apoptosis to necrosis in *Caenorhabditis elegans* (Syntichaki et al., 2005). Depending on the extracellular pH, cells invest significant amounts of energy to keep intracellular pH homeostasis to maintain viability (Della-Bianca et al., 2014). Increased energy expenditure in acidified non-controlled cultures negatively influences viability, as other studies showed that CLS can be enhanced by buffering the culture pH (Fabrizio et al., 2004). And these effects might be even stronger in already challenged Aβ42 expressing cells. In addition to contributing to low pH, organic acids have been shown to affect cell performance in specific manners (Abbott et al., 2007). Especially acetic acid is a potent cell-stressor that has been shown to induce lysosomal apoptotic pathway either in a mitochondria-independent or dependent way (Marques et al., 2013; Oliveira et al., 2015). Under bioreactor conditions less acetate was produced by all strains than in shake-flask cultures (Figure S4 and Table 1). Higher initial glucose concentrations also led to higher acetate levels, especially for the Aβ42 strain. Overall the reduced oxygen availability, low pH, and increased acetate levels cause additional stress and strongly affect survival of Aβ42 expressing cells.

Human brain activity corresponds to a high fraction of total energy consumption, and neurons strongly depend on mitochondria due to a limited glycolytic capacity (Moreira et al., 2009). Mitochondrial dysfunction leads to energy metabolism abnormalities that endanger normal neuron functioning and contribute to AD pathology. In yeast expressing Aβ42, lower growth rates and reduced biomass yields were observed (Table 1). This reflects a redirection of energy from growth to maintenance, i.e., processes aimed at maintaining cell integrity and functioning that do not lead to an increase in biomass. In addition, it also points at reduced ATP generation from the energy sources consumed as indicated by reduced oxygen consumption rates and lower respiratory quotient (Figure 2A and Table S1). Reduced energy metabolism in the diseased brain is one of best documented abnormalities in AD (Moreira et al., 2007). In fact, the low glucose baseline metabolism and its decline during aging are viewed as sensitive measures and being increasingly adopted to assist diagnosis in cognitive decline (Shokouhi et al., 2013; Yamane et al., 2014). Several groups reported that Aβ peptides accumulate in mitochondria and directly interact with several mitochondrial proteins (Manczak et al., 2006; Pagani and Eckert, 2011; Pavlov et al., 2011). The interaction of Aβ peptides with Aβ-binding alcohol dehydrogenase (ABAD) in mitochondria promotes leakage of ROS, mitochondrial dysfunction and cell death in AD patients and transgenic mice (Lustbader et al., 2004). The interaction of Aβ peptides with mitochondria appears to affect a multitude of different functions in AD, including respiration, detoxification of ROS, and organellar morphology (Rhein et al., 2009; Yao et al., 2009; Selfridge et al., 2013). Here, we observed similar responses specifically in yeast cells expressing the more toxic Aβ42 peptide. With TPEF microscopy, aberrant fragmented mitochondrial structures were detected in the Aβ42 strain. In brain tissue from AD patients and neuronal cells expressing mutant APP, similar fragmented mitochondria and structural changes have been observed (Hirai et al., 2001; Wang et al., 2008).

Genome wide expression level analysis revealed that both Aβ peptides expression induced ESR, although the Aβ40 strain did not show significant physiological changes (Figure 3B). Studies proposed an important role for ER stress in AD pathogenesis by acting as a mediator of Aβ neurotoxicity (Umeda et al., 2011; Hoozemans et al., 2012). Aβ furthermore triggers ER stress-specific apoptosis through caspase-12 and caspase-4 (Nakagawa et al., 2000; Hitomi et al., 2004). ER stress results in activation of UPR, one of stress response pathways, which aims to restore ER homeostasis (Cao and Kaufman, 2012). In yeast, the UPR is regulated solely by the Ire1 pathway which is conserved from yeast to mammals (Iwawaki et al., 2001). In response to ER stress, activated Ire1p splices *HAC1*^*u*^ to *HAC1*^*s*^ to initiate synthesis of Hac1p which translocates into nucleus to regulate expression of UPR target genes (Mori et al., 2000). The different ratio of *HAC1*^*s*^/*HAC1*^*u*^ in Aβ42 and Aβ40 strains suggested that the expression of these variants results in a different extent of ER stress and consequently UPR. This is in accordance with previous findings in yeast, which show that UPR signaling can be modulated through differential target gene expression depending on the nature of stress (Thibault et al., 2011). The different nature of the two Aβ peptides investigated here, showed that Aβ40 triggered a mild response, and Aβ42 resulted in a stronger stress affecting many aspects of the physiology of the cells. Decreasing the ER protein load is the first attempt of UPR to restore proteostasis. In our study, the processes involved in protein folding/maturation, ER-to-Golgi trafficking and ERAD were significantly upregulated in both Aβ strains initiating from EX, though at different levels. In addition to ERAD, autophagy was activated only in the Aβ42 strain, which suggested that the amounts of misfolded or aggregated proteins exceed the ER capacity (Figure 4). Global transcription, translation, and amino acid synthesis were repressed in response to Aβ42 expression (Figure 3B and Figure S7). By these responses, the influx of new proteins into ER can be reduced, whereas the efflux is increased. The large fraction of nuclear-DNA encoded mitochondrial proteins is processed through the ER. Reduced mitochondrial protein biogenesis and turn-over can result in increased dysfunction, thereby linking the UPR with energy metabolism. Mitochondria furthermore communicate directly with ER through MAM (mitochondria-associated ER membranes) to regulate several fundamental cellular processes (Csordás et al., 2006; Rowland and Voeltz, 2012). This crosstalk between ER and mitochondria may have a role in facilitating stress response and UPR (Bernales et al., 2012; Stoica et al., 2014; Paillusson et al., 2016). However, these processes were up-regulated in the Aβ40 strain during PD, which suggested the cells started to re-establish homeostasis within the ER (Figure 3B and Figure S8). When the buffering capacity of UPR proves inadequate to restore ER proteostasis, the pathway switches from an adaptation program to apoptosis to remove irreversibly damaged cells (Rutkowski et al., 2006; Szegezdi et al., 2006). This is reflected by the significantly elevated fractions of dead cells in Aβ42 strain cultures, compared to cultures of control and Aβ40 strains (Figure S2A).

Recently, it was found that ER stress and UPR activation regulate cellular processes beyond ER protein folding and play crucial roles in lipid metabolism by controlling the transcriptional regulation of lipogenesis in the liver (Lee et al., 2008; Zhang et al., 2011). We found that at the transcript level, genes involved in lipid biosynthesis were higher expressed in both Aβ strains compared to the control strain during EX (Figure 5A). UPR regulates inositol, an important regulator of lipid metabolism in yeast, which plays a key role in phospholipid biosynthesis required for membranes (Jesch et al., 2005). The expression levels of *INO1*, the gene encoding the enzyme that catalyzes the rate-limiting step in *de novo* synthesis of inositol (Henry et al., 2012), was significantly increased in the Aβ42 strain during EX. Moreover, the tight link between lipid synthesis and UPR was shown previously by the induction of UPR upon *OPI3* or *INO1* deletion (Jonikas et al., 2009). We used lipidomics to quantify the lipid and sterol components and it showed a significant increase in phospholipids and storage lipids during growth phases in the Aβ42 strain (Figure 5B). This result was further supported by CARS microscopy, which showed significantly larger lipid drops in the Aβ42 strain compared to the control strain during EX (Figure 5C). Links between lipid metabolism and AD have been previously proposed, since the initial observation that feeding rabbits with a cholesterol-enriched diet leads to Aβ accumulation (Sparks et al., 1994). Nowadays several studies provide substantial evidence that aberrant lipid metabolism is closely connected to Aβ modulation during the pathogenesis of AD in humans (Wood, 2012; Walter and van Echten-Deckert, 2013). Apolipoprotein E (ApoE) is the strongest known genetic risk factor for the most common late-onset sporadic AD (Corder et al., 1993; Strittmatter et al., 1993), which might impair Aβ clearance and increase its aggregation in the brain (Bales et al., 1997; Holtzman et al., 2000; Verghese et al., 2013). Moreover, alterations in membrane lipid composition, including cholesterol and sphingolipids may also affect Aβ generation and aggregation properties (Simons et al., 1998; Sawamura et al., 2004). Conversely, Aβ can influence lipid homeostasis by modulating lipid metabolic enzymes and directly binding to membrane lipids (Cutler et al., 2004; Grimm et al., 2005). Our study demonstrated that Aβ-induced ER stress might be an additional mechanism contributing to the increased brain cholesterol content observed in AD.

**Figure 6:**
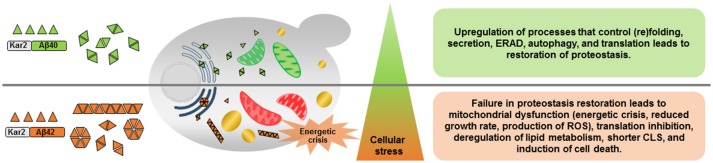
Schematic overview of the effects of constitutive expression of Aβ40 or Aβ42 peptides. Aβ40 and Aβ42 differ in potential to form aggregates resulting in different levels of ER stress and induction of the unfolded protein response (UPR). Constitutive expression of Aβ40, induces mild ER stress and subsequent activation of the UPR recovers proteome homeostasis (proteostasis) by promoting protein (re)folding, protein quality control, and degradation mechanisms. Aβ42 peptides, on the contrary, result in prolonged ER stress and the strongly activated UPR fails to buffer the misfolded protein load, leading to cellular dysfunction and a shorter chronological life span (CLS). ERAD, ER-associated degradation; ROS, reactive oxygen species; Green mitochondria, functional; Red mitochondria, dysfunctional; Light blue ER, mild ER stress; Dark blue ER, strong ER stress; Yellow, lipid droplets.

Overall, this study provided highly informative data regarding changes in cellular metabolic activity and energy metabolism as consequences of different Aβ peptides expression. The expression of Aβ40 and Aβ42 peptides also caused different levels of ER stress which tightly regulates the amplitude and kinetics of UPR signaling to decide different cell fates (Figure 6). Due to the emerging role of UPR in diverse disease conditions, such as, cancer, diabetes, and neurodegeneration, understanding how the UPR interacts with other cellular regulations is fundamental for the identification of future points of intervention in many important and to date often incurable human diseases.

## Author contributions

XC, MB, and DP designed research; XC and MB performed and analyzed batch cultivation, extracellular metabolites, mitochondrial bioenergetics, molecular, and cellular experiments. NA performed Non-linear microscopy experiments. KS prepared cells for Non-linear microscopy experiments. XC, MB, and BJ analyzed microarray data. XC, MB, and DP wrote the manuscript, with contributions from NA and BJ.

### Conflict of interest statement

The authors declare that the research was conducted in the absence of any commercial or financial relationships that could be construed as a potential conflict of interest.

## Supplementary material

Figure S1 Cellular reserve carbohydrate concentrations in batch cultures. Cellular contents of glycogen **(A)** and trehalose **(B)** are measured during PD, SP1, and SP2 phases under aerobic condition. Results represent average values ± SEM, of triplicate (Aβ42 and control) or duplicate (Aβ40) independent biological replicates. The asterisk (^*^) indicates significant differences (*p* < 0.001).

Figure S2 Fractions of dead cells and reactive oxygen species (ROS) positive cells as function of age in aerobic and microaerobic cultures. **(A)** Cells were stained with PI and analyzed by flow cytometry, PI-positive cells are considered dead. **(B)** Cells were stained with DHR123 and analyzed by flow cytometry. Strongly fluorescent cells (i.e., 5–100 times more fluorescent than low fluorescent cells) are considered ROS positive. Insert shows the values for the first three growth phases in detail. Full lines and dotted lines indicate aerobic or microaerobic conditions, respectively.

Figure S3 Comparison of fractions of dead cells **(A,B)**, pH **(C,D)**, and cell growth **(E,F)** between 1 and 2% glucose cultures grown in shake flasks.

Figure S4 Quantification and comparison of acetate production under different culture conditions after glucose exhaustion (diauxic shift, DS).

Figure S5 Principle Component Analysis (PCA). Histogram of variance for each PC shows that the first two PCs capture the largest variance of dataset, which are 70% (PC1) and 19.8% (PC2), respectively.

Figure S6 The Venn diagrams show distribution of significantly differentially expressed genes between Aβ42 and control strains **(A)**, Aβ40 and control strains **(B)**, Aβ42 and Aβ40 strains **(C)** during different growth phases (*p* < 0.001).

Figure S7 The significantly enriched GO terms in Aβ42 strain among genes differentially expressed compared to control strain (*p* < 0.001).

Figure S8 The significantly enriched GO terms in Aβ40 strain among genes differentially expressed compared to control strain (*p* < 0.001).

Figure S9 Schematic overview of significantly changed genes in amino acid biosynthetic pathways in Aβ42 strain **(A)** and Aβ40 strain **(B)** compared to control strain. Differences in gene expression levels are shown as fold changes compared to control strain during EX, PD, SP1, and SP2 phases (*p* < 0.05).

Figure S10 qPCR analysis of *HAC1*^*s*^/*HAC1*^*u*^ ratio **(A)**, *PDI1* **(B)**, and *ERO1* **(C)** mRNA levels in all strains from EX, PD, and SP1 phases. Results are average values ± SEM, of triplicate (Aβ42 and control) or duplicate (Aβ40) independent biological replicates. The asterisk (^*^) indicates significant differences (*p* < 0.05).

Figure S11 Transcriptional profiles of differentially expressed genes related to protein secretory and metabolic processes in Aβ40 strain compared to control strain. Changes in gene expressions are shown as fold changes compared to control strain during EX, PD, SP1, and SP2.

Figure S12 The Venn diagrams show distribution of significantly differentially expressed genes in protein secretory and metabolic processes between Aβ42 and Aβ40 strains during EX **(A)**, PD **(B)**, and SP1 **(C)** phases (*p* < 0.05).

Figure S13 Quantitative analysis of CARS microscopy images presenting the contents of lipids in control and Aβ42 expressing cells during EX. Ten images were analyzed for each strain. Each image contains one or more cells. The value of lipid content was calculated by the ratio of summation over stacks of lipids area/summation over stacks of cell area. The average lipid content was higher in Aβ42 strain (0.078 ± 0.032 μm^3^) than in control strain (0.048 ± 0.028 μm^3^).

Table S1 Physiological parameters of micro-aerobic batch cultures.

Table S2 Genes with significantly different expression in amino acid biosynthetic pathways (Aβ42 strain vs. control strain).

Table S3 Genes with significantly different expression in protein secretory and metabolic processes (Aβ40 strain vs. control strain).

Table S4 Primer-sets used for qPCR.

Video S1 3D movie of a representative image of mitochondrial structure in control cell. Mitochondria were stained with Rhodamine 123 and shown in green. Red shows the background of TPEF microscopy and is indicative of cell volume.

Video S2 3D movie of a representative image of mitochondrial structure in Aβ42-expressing cells. Mitochondria were stained with Rhodamine 123 and shown in green. Red shows the background of TPEF microscopy and is indicative of cell volume.

Video S3 3D movie of a representative image of lipid drops in control cell. Yellow, lipid drops; red, background of CARS microscopy to indicate the cell volume.

Video S4 3D movie of a representative images of lipid drops in Aβ42-expressing cells. Yellow, lipid drops; red, background of CARS microscopy to indicate the cell volume.
