# Supplementary material for: Interplay of Energetics and ER Stress Exacerbates Alzheimer's Amyloid-β (Aβ) Toxicity in Yeast
Source: Front Mol Neurosci. 2017 Jul 27;10:232. doi: 10.3389/fnmol.2017.00232 (PMC5529408; doi:10.3389/fnmol.2017.00232)
Supplement: Figure S1 — Cellular reserve carbohydrate concentrations in batch cultures. Cellular contents of glycogen (A) and trehalose (B) are measured during PD, SP1, and SP2 phases under aerobic condition. Results represent average values ± SEM, of triplicate (Aβ42 and control) or duplicate (Aβ40) independent biological replicates. The asterisk (*) indicates significant differences (p < 0.001). [file Image1.pdf]

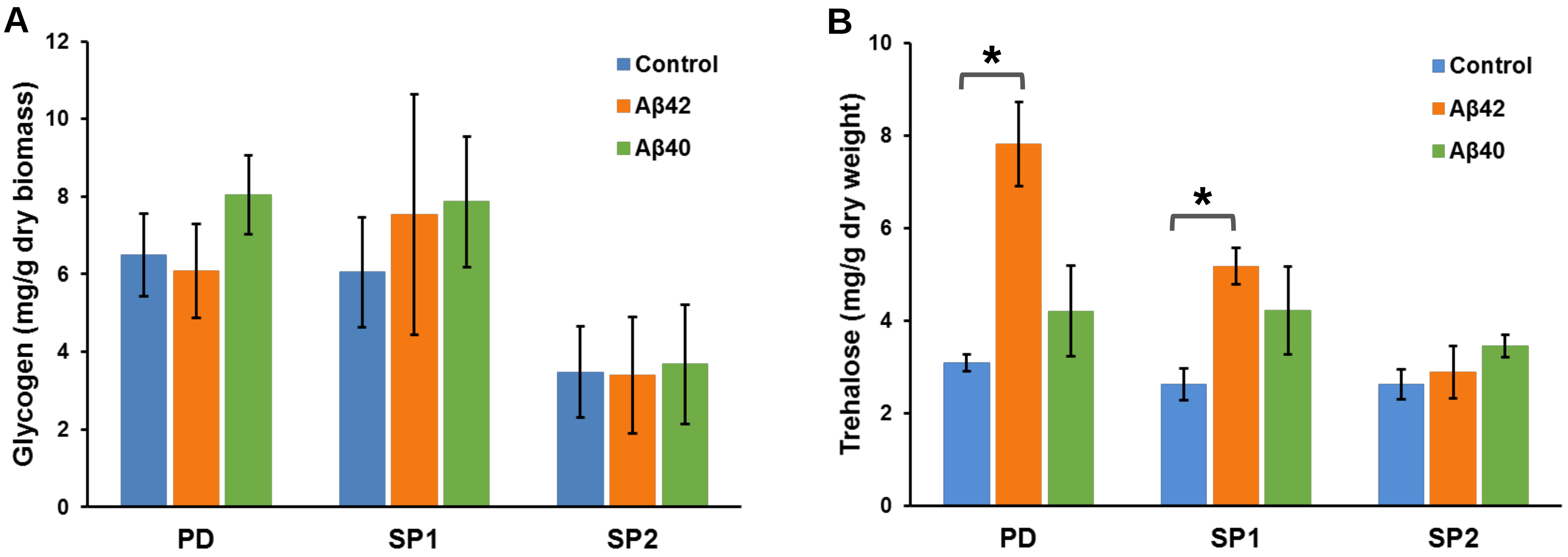


Fig. S1. Cellular reserve carbohydrate concentrations in batch cultures. Cellular contents of glycogen (A) and trehalose (B) are measured during PD, SP1 and SP2 phases under aerobic condition. Results are representative of average values ± SEM, of triplicate (Aβ42 and control) or duplicate (Aβ40) independent biological replicates. The asterisk (*) indicates significant differences ($p$ < 0.001).

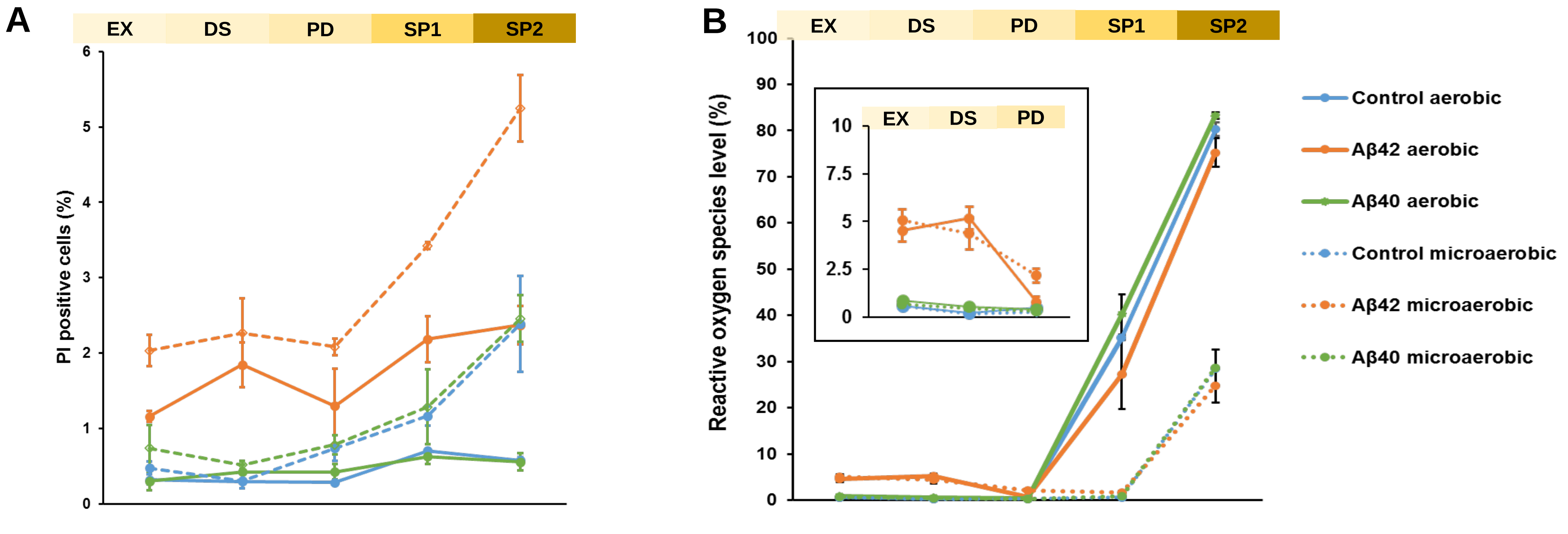


Fig. S2. Fractions of dead cells and reactive oxygen species (ROS) positive cells as function of age in aerobic and microaerobic cultures. (A) Cells were stained with PI and analysed by flow cytometry, PI-positive cells are considered dead. (B) Cells were stained with DHR123 and analysed by flow cytometry. Strongly fluorescent cells (i.e., 5 to 100 times more fluorescent than low fluorescent cells) are considered ROS positive. Insert shows the values for the first three growth phases in detail. Full lines and dotted lines indicate aerobic or microaerobic conditions respectively.

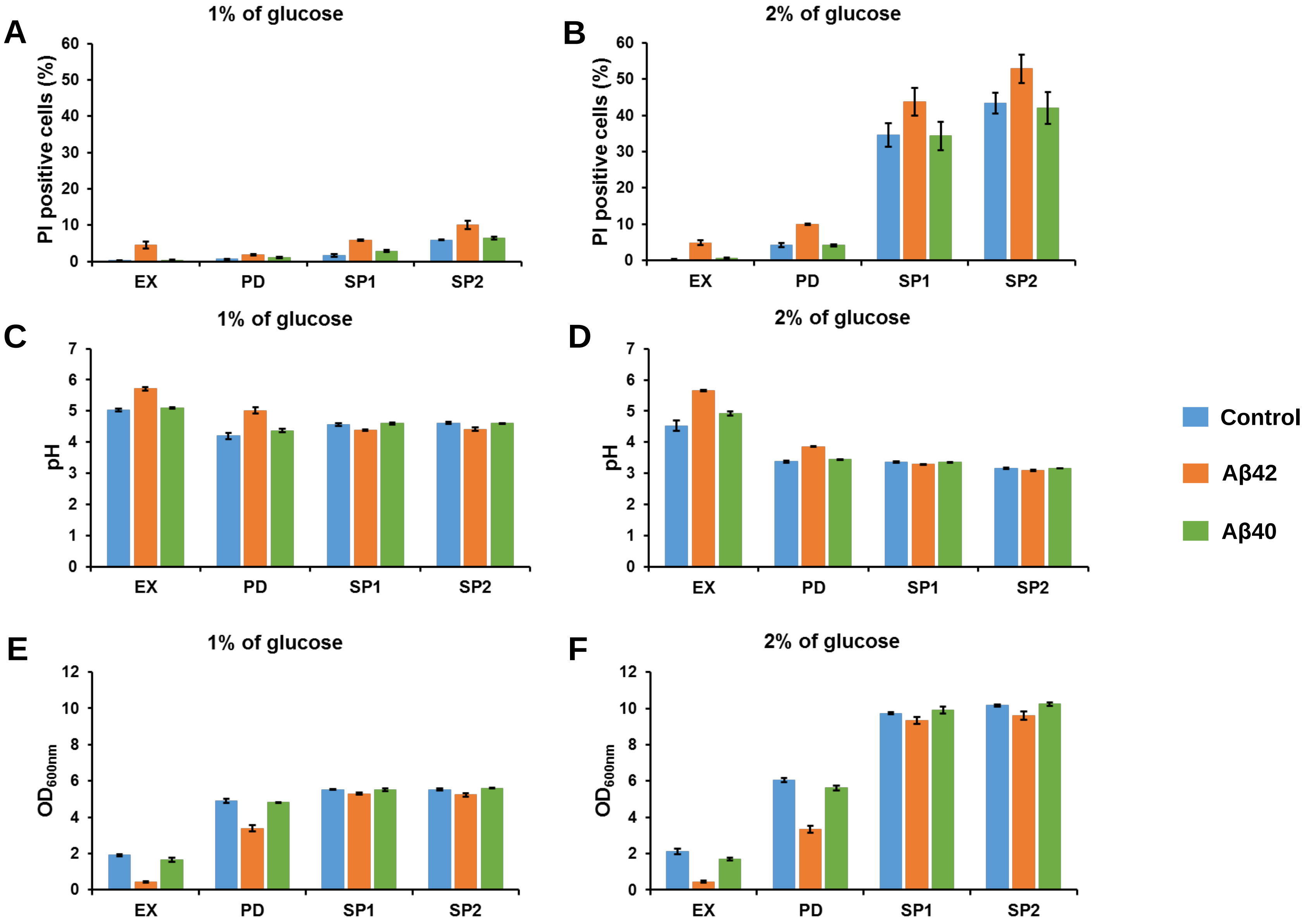


Fig. S3. Comparison of fractions of dead cells (A and B), pH (C and D) and cell growth (E and F) between 1% and 2% glucose cultures grown in shake flasks.

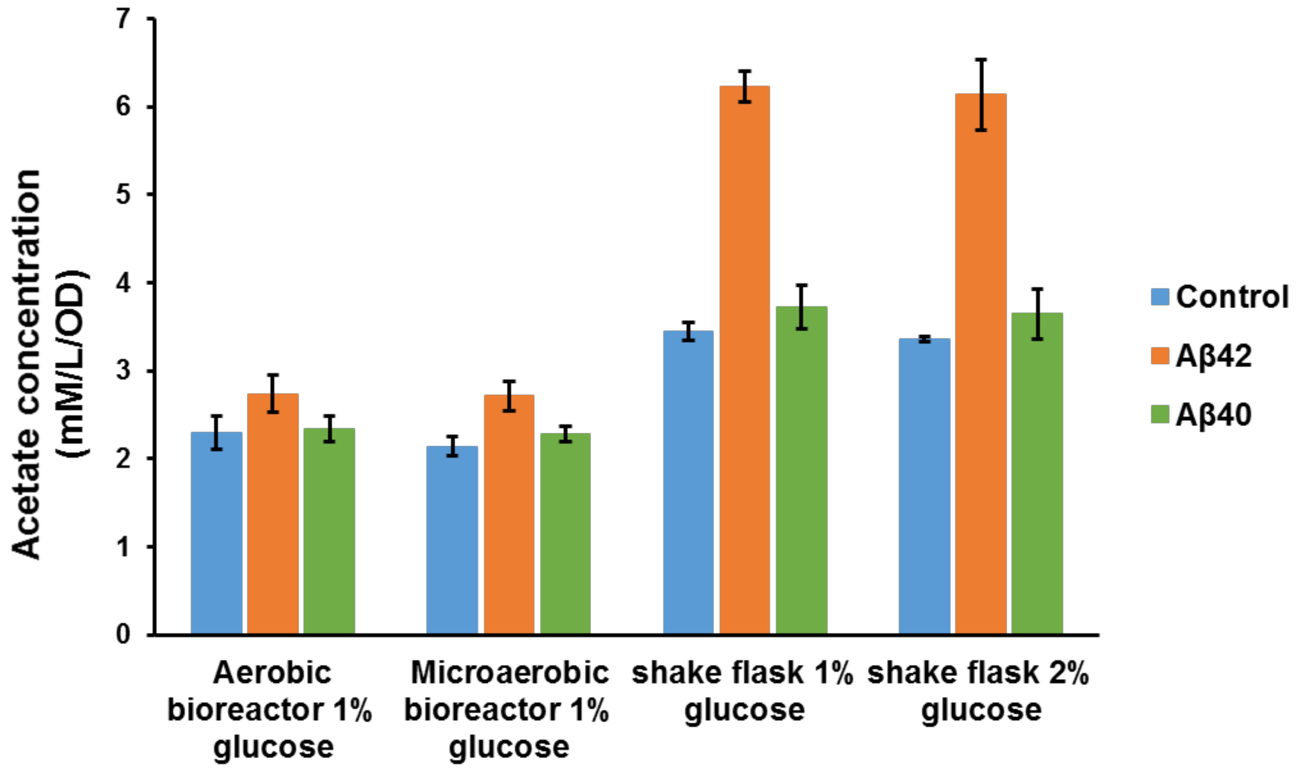


Fig. S4. Quantification and comparison of acetate production under different culture conditions after glucose exhaustion (diauxic shift, DS).

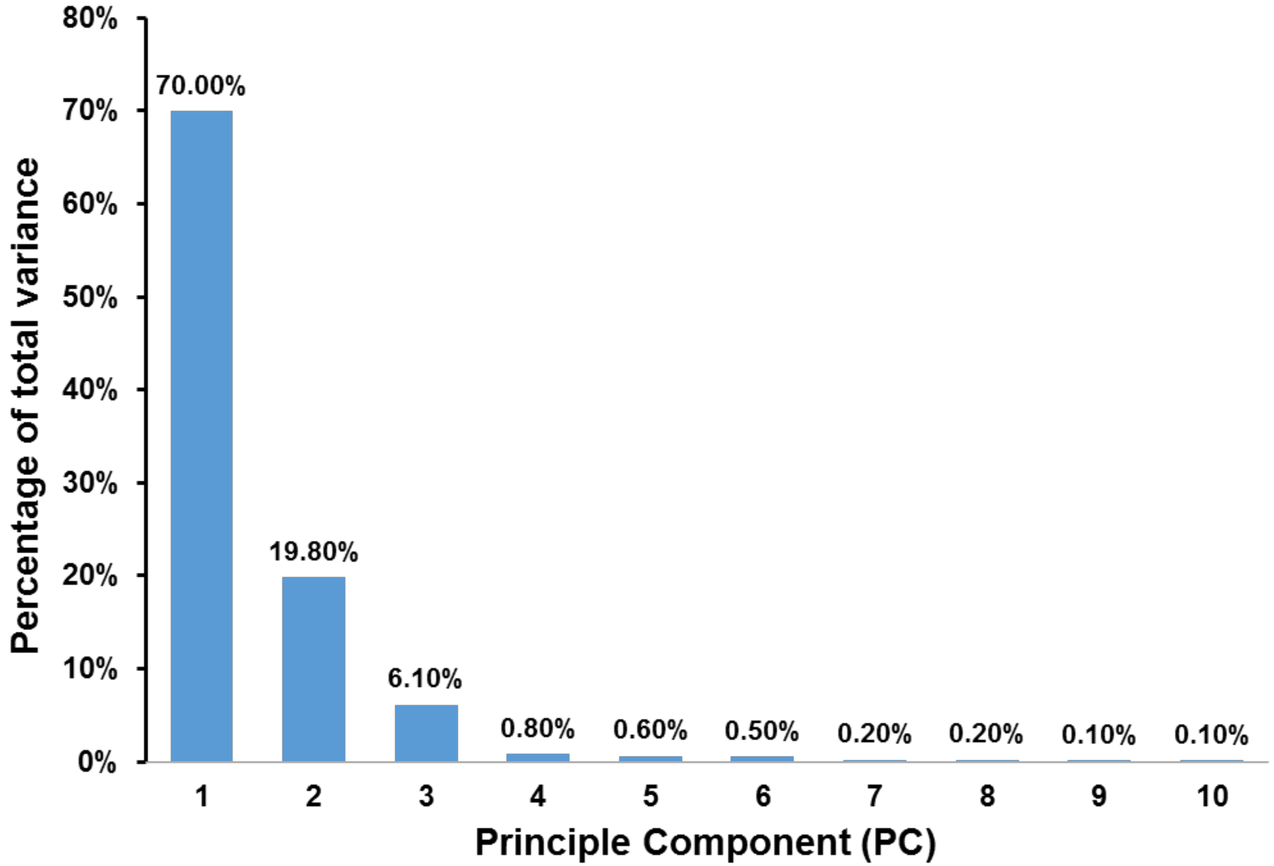


Fig. S5. Principle Component Analysis (PCA). Histogram of variance for each PC shows that the first two PCs capture the largest variance of dataset, which are 70% (PC1) and 19.8% (PC2) respectively.

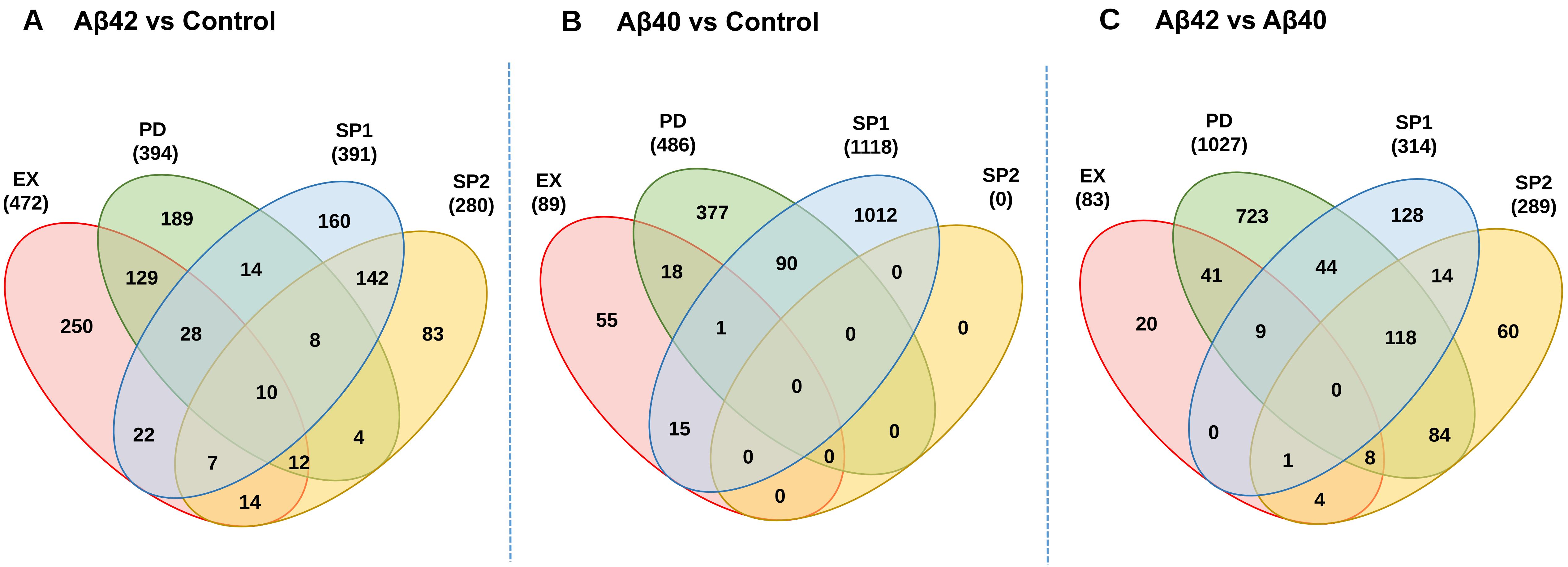


Fig. S6. The Venn diagrams show distribution of significantly differentially expressed genes between Aβ42 and control strains (A), Aβ40 and control strains (B), Aβ42 and Aβ40 strains (C) during different growth phases ($p < 0.001$).

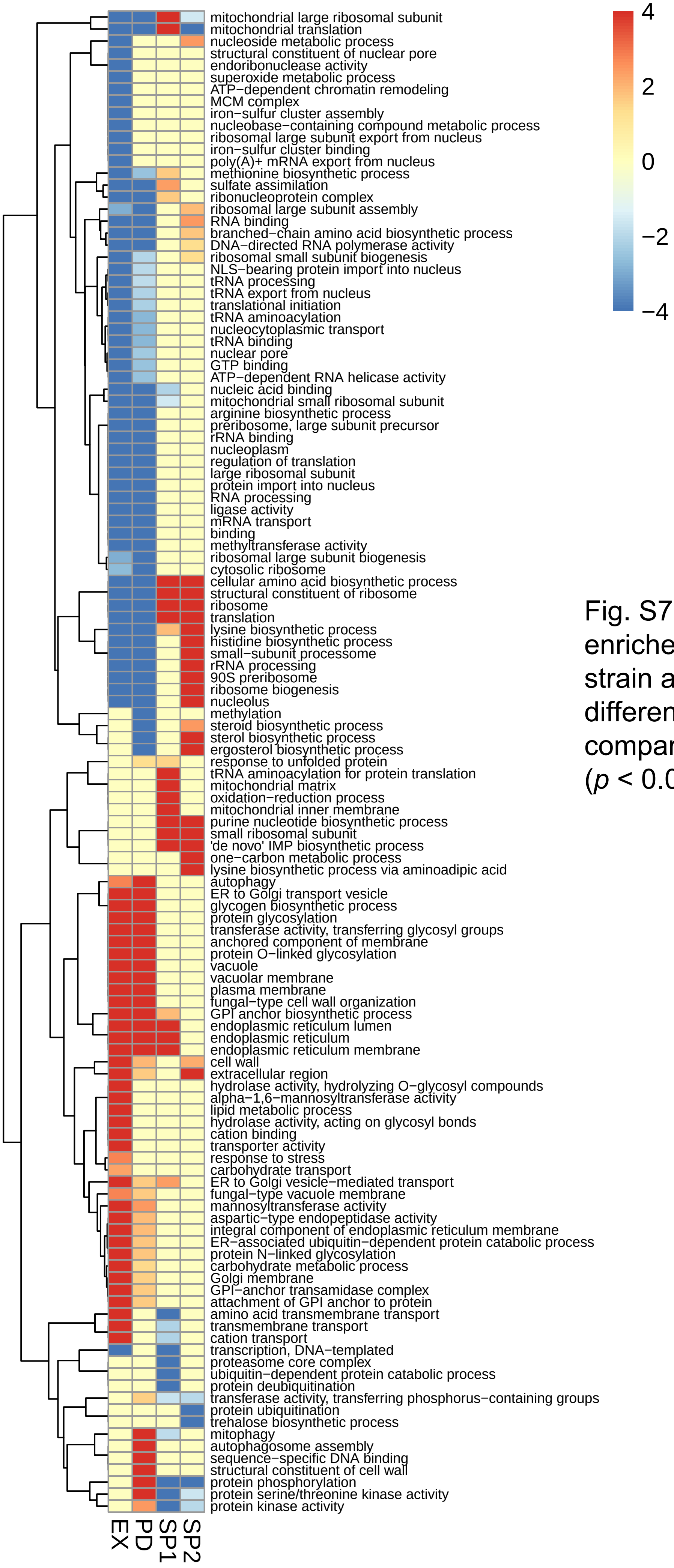


Fig. S7. The significantly enriched GO terms in Aβ42 strain among genes differentially expressed compared to control strain ($p$ < 0.001).

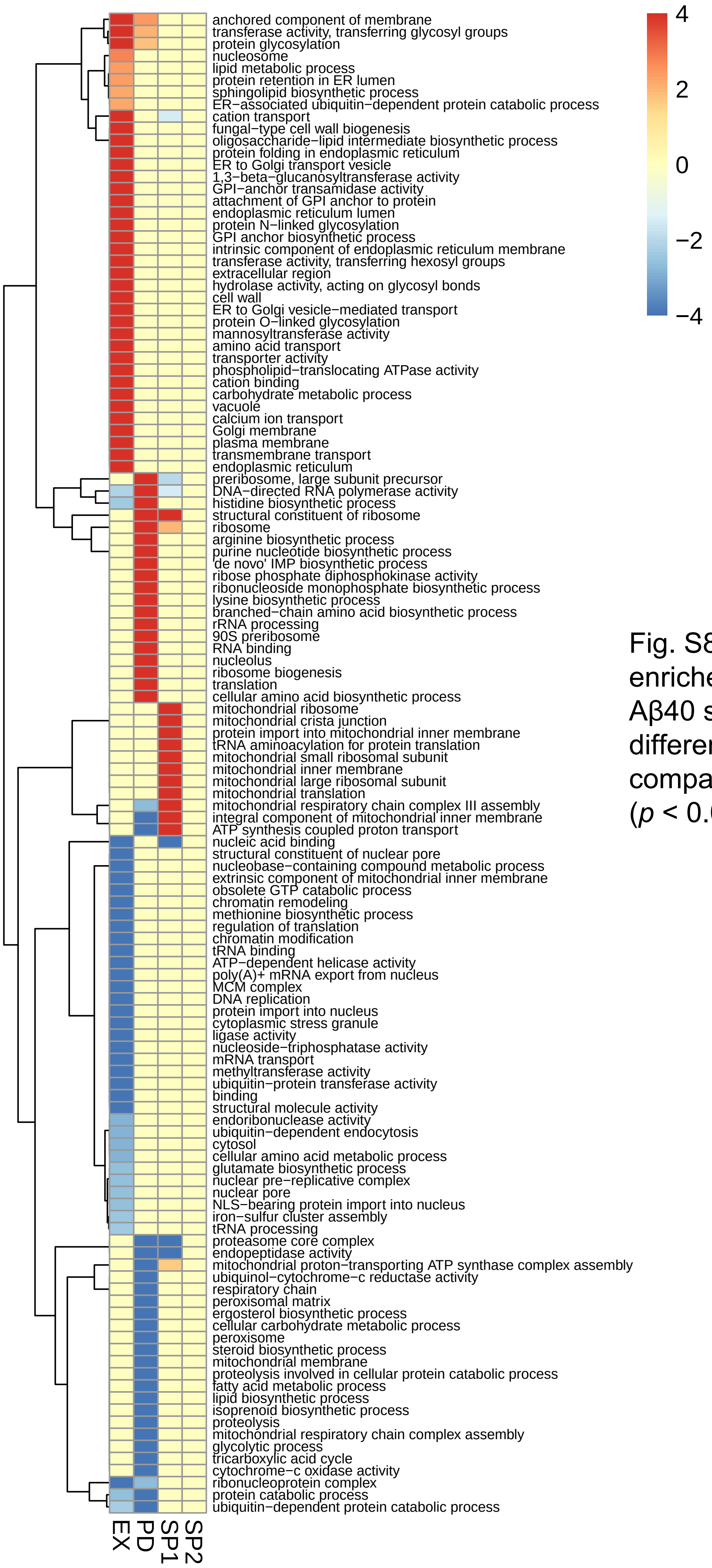


Fig. S8. The significantly enriched GO terms in Aβ40 strain among genes differentially expressed compared to control strain ($p < 0.001$).

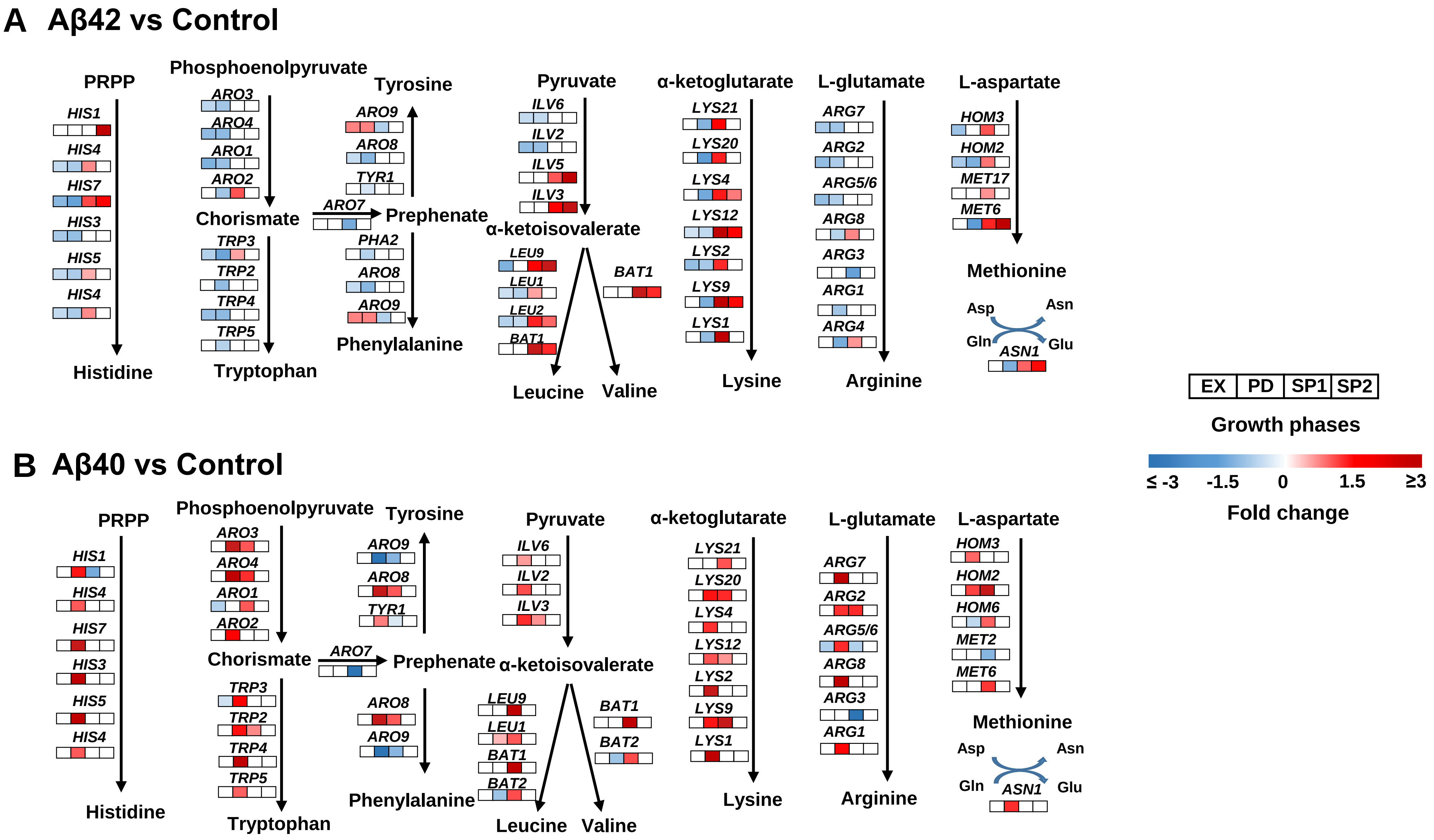


Fig. S9. Schematic overview of significantly changed genes in amino acid biosynthetic pathways in Aβ42 strain (A) and Aβ40 strain (B) compared to control strain. Differences in gene expression levels are shown as fold changes compared to control strain during EX, PD, SP1 and SP2 phases ($p < 0.05$).

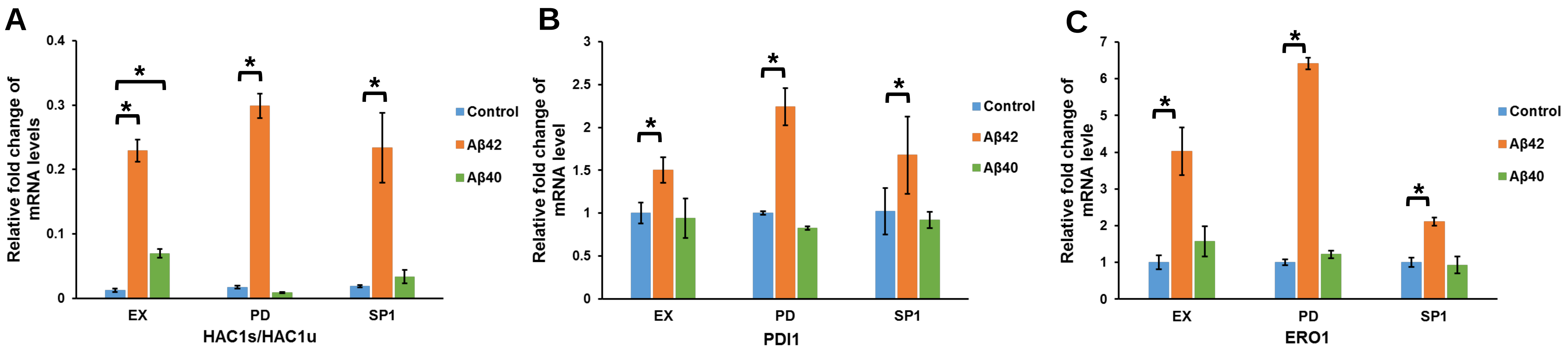


Fig. S10. qPCR analysis of *HAC1^s^/HAC1^u^* ratio (A), PDI1 (B) and EROI (C) mRNA levels in all strains from EX, PD and SP1 phases. Results are average values ± SEM, of triplicate (Aβ42 and control) or duplicate (Aβ40) independent biological replicates. The asterisk (*) indicates significant differences ($p < 0.05$).

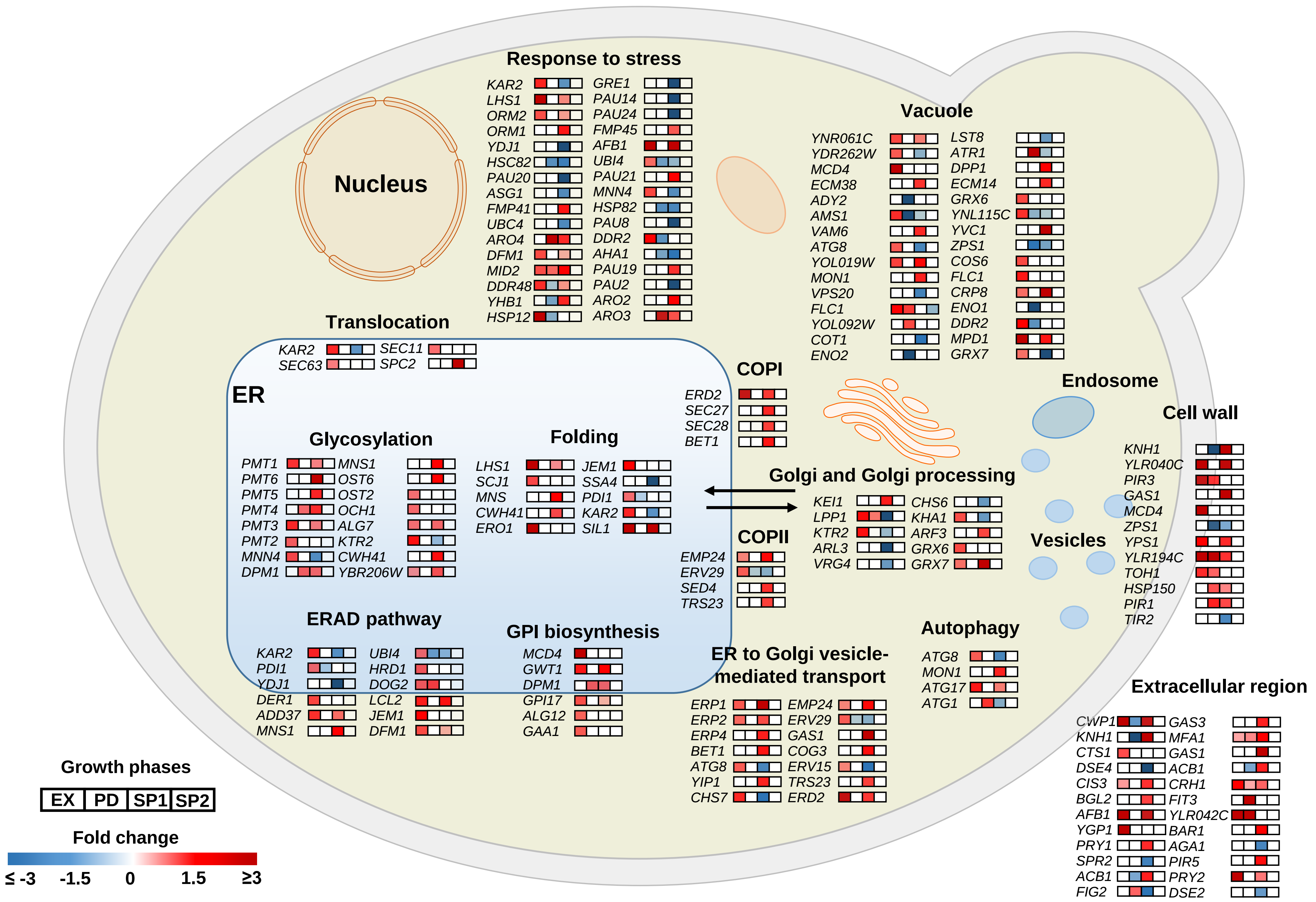


Fig. S11. Transcriptional profiles of differentially expressed genes related to protein secretory and metabolic processes in Aβ40 strain compared to control strain. Changes in gene expressions are shown as fold changes compared to control strain during EX, PD, SP1 and SP2.

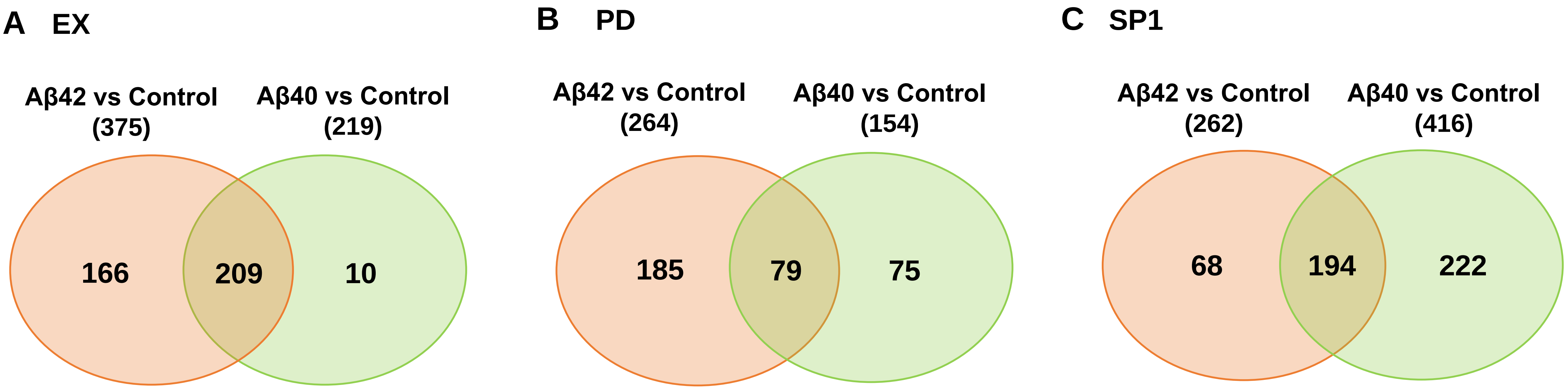


Fig. S12. The Venn diagrams show distribution of significantly differentially expressed genes in protein secretory and metabolic processes between Aβ42 and Aβ40 strains during EX (A), PD (B) and SP1 (C) phases ($p < 0.05$).

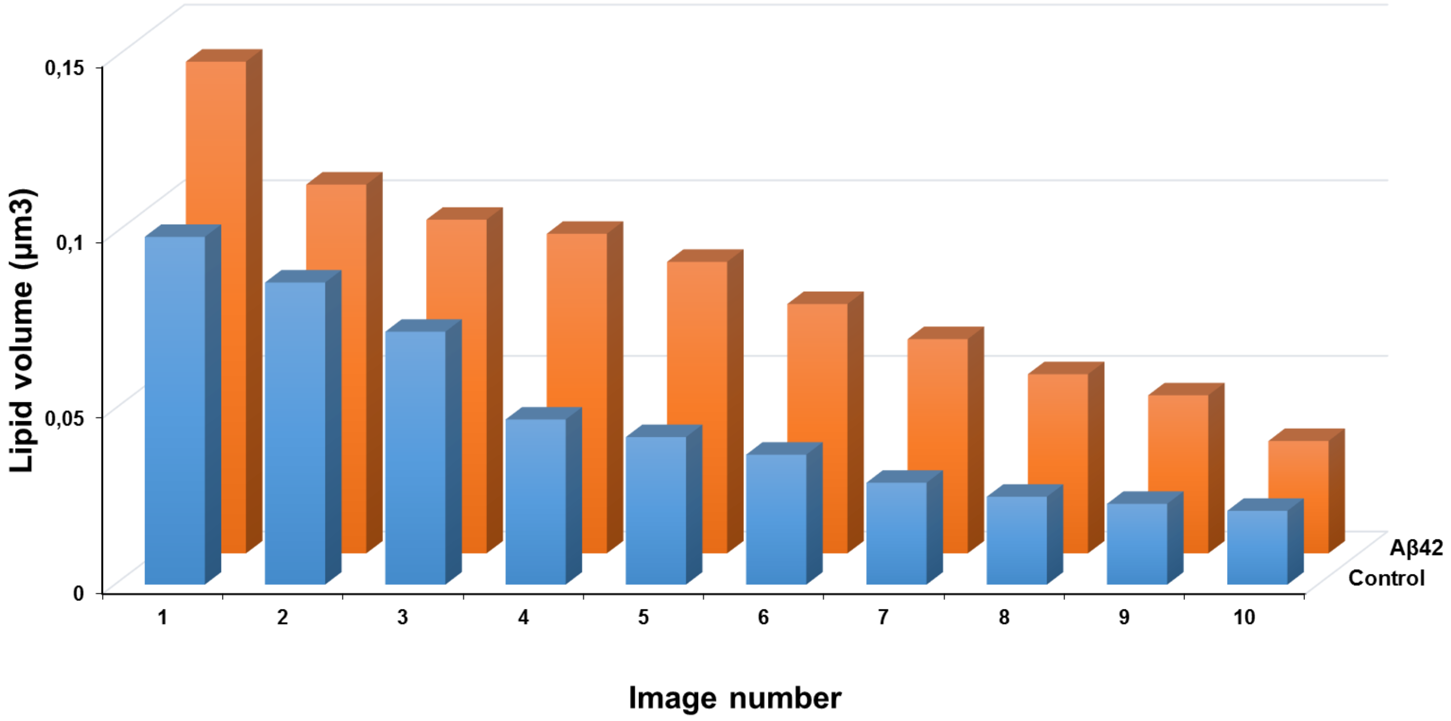


Fig. S13. Quantitative analysis of CARS microscopy images presenting the contents of lipids in control and Aβ42 expressing cells during EX. 10 images were analyzed for each strain. Each image contains one or more cells. The value of lipid content was calculated by the ratio of summation over stacks of lipids area / summation over stacks of cell area. The average lipid content was higher in Aβ42 strain (0.078±0.032 $\mu m^3$) than in control strain (0.048±0.028 $\mu m^3$).
