# Supplementary material for: Interplay of Energetics and ER Stress Exacerbates Alzheimer's Amyloid-β (Aβ) Toxicity in Yeast
Source: Front Mol Neurosci. 2017 Jul 27;10:232. doi: 10.3389/fnmol.2017.00232 (PMC5529408; doi:10.3389/fnmol.2017.00232)
Supplement: Table S1 — Physiological parameters of micro-aerobic batch cultures. [file Table1.pdf]

Table S1. Physiological parameters of micro-aerobic batch cultures.

Strain	μ_{max} [a] (/h)	$Y_{X/S}$ [b] (g/g)	Respiratory quotient[c]	$r_{O2,max}$ [d] (mmol/g/h)
**Control**	0.372 ± 0.005	0.39 ± 0.01	0.46 ± 0.04	2.9 ± 0.5
**Aβ40**[e]	0.359 ± 0.005	0.352 ± 0.003	0.41 ± 0.05	3.1 ± 1.0
**Aβ42**	0.280* ± 0.009	0.33* ± 0.01	0.33* ± 0.05	3.3 ± 0.5

Values are represented as the average of three independent biological replicates ± SEM. * indicates significant different values from the control strain parameters ($p < 0.05$). a Maximal biomass-specific growth rate on glucose; b Final biomass yield on substrate; c Respiratory quotient during PD; d Maximal biomass-specific oxygen uptake rate during PD; e The average values from biological duplicate cultivations.
