# Supplementary material for: Interplay of Energetics and ER Stress Exacerbates Alzheimer's Amyloid-β (Aβ) Toxicity in Yeast
Source: Front Mol Neurosci. 2017 Jul 27;10:232. doi: 10.3389/fnmol.2017.00232 (PMC5529408; doi:10.3389/fnmol.2017.00232)
Supplement: Table S4 — Primer-sets used for qPCR. [file Table4.pdf]

Table S4. Primer-sets used for qPCR.

Gene	Primer (FR & BR)
$HAC1^{U}$	5’-CAATTGGCGTAATCCAGCCG-3’
	5’-AGCTGGGGCTAGTGTTCTTG-3’
$HAC1^{S}$	5’-GCGTCGGACCAAGAGACTTC-3’
	5’-CTGACTGCGCTTCTGGATTAC-3’
PDI1	5’-CCCAGGTGGTAAGAAGTCCG-3’
	5’-CAATTCAGCGTCAGCATCGG-3’
ERO1	5’-CGCTTGCTCTGTTGATGTCG-3’
	5’-TCGTCATCGCTATCATCCGC-3’
ACT1	5’-AGTTGCCCCAGAAGAACACC-3’
	5’-GGACAAAACGGCTTGGATGG-3’
